# Differential Proteomic Analysis of Lactic Acid Bacteria—*Escherichia coli* O157:H7 Interaction and Its Contribution to Bioprotection Strategies in Meat

**DOI:** 10.3389/fmicb.2018.01083

**Published:** 2018-06-05

**Authors:** Alejandra Orihuel, Lucrecia Terán, Jenny Renaut, Graciela M. Vignolo, André M. De Almeida, María L. Saavedra, Silvina Fadda

**Affiliations:** ^1^Technology Department, Centro de Referencia para Lactobacilos, Consejo Nacional de Investigaciones Científicas y Técnicas (CERELA CONICET), San Miguel de Tucumán, Argentina; ^2^Genetics and Molecular Biology Department, Centro de Referencia para Lactobacilos, Consejo Nacional de Investigaciones Científicas y Técnicas (CERELA CONICET), San Miguel de Tucumán, Argentina; ^3^Luxembourg Institute of Science and Technology “Environmental Research and Innovation” Department, Belvaux, Luxemburg; ^4^LEAF - Linking Landscape, Environment, Agriculture and Food, Instituto Superior de Agronomia, University of Lisbon, Lisbon, Portugal

**Keywords:** Lactic acid bacteria (LAB), bioprotective cultures, enterohemorrhagic *Escherichia coli* (EHEC), meat safety, bacterial interaction, proteomics

## Abstract

Human infection by Enterohemorrhagic *Escherichia (E.) coli* (EHEC) occurs through the ingestion of contaminated foods such as milk, vegetable products, water-based drinks, and particularly minced meats. Indeed EHEC is a pathogen that threatens public health and meat industry. The potential of different Lactic Acid Bacteria (LAB) strains to control EHEC in a meat-based medium was evaluated by using a simple and rapid method and by analyzing the growth kinetics of co-cultures (LAB-EHEC) in a meat-based medium. The activity of LAB toward EHEC in co-cultures showed variable inhibitory effect. Although, LAB were able to control EHEC, neither the produced acid nor bacteriocins were responsible of the inhibition. The bacteriocinogenic *Enteroccus (Ent.) mundtii* CRL35 presented one of the highest inhibition activities. A proteomic approach was used to evaluate bacterial interaction and antagonistic mechanisms between *Ent. mundtii* and EHEC. Physiological observations, such as growth kinetics, acidification ability and EHEC inhibitory potential were supported by the proteomic results, demonstrating significant differences in protein expression in LAB: (i) due to the presence of the pathogen and (ii) according to the growth phase analyzed. Most of the identified proteins belonged to carbohydrate/amino acid metabolism, energy production, transcription/translation, and cell division. These results contribute to the knowledge of competition strategies used by *Ent. mundtii* during its co-culture with EHEC setting new perspectives for the use of LAB to control this pathogen in meat.

## Introduction

Contamination with Shiga toxin-producing *Escherichia (E.) coli* (STEC) and related enteric pathogens is among the main causes of concern and fresh meat product recalls. In the European Union STEC prevalence on hides is estimated at 44%, before falling to 0.4% on carcasses, and 1.2% in raw beef meat. In addition, in the United States, the Centers for Disease Control and Prevention (CDC) have estimated that STEC infections cause 73,000 illnesses, 2,200 hospitalizations, and 60 deaths yearly. The annual cost of illness due to STEC was 405 million dollars, including lost productivity, medical care, and premature deaths (Lim et al., 2010). High economic losses in meat industry and the high cost of the illness evidence the necessity of additional efforts to control this pathogen. Within the STEC pathotype, the *E. coli* enterohemorrhagic (EHEC) subgroup is important because of its impact on Public Health. Human infection by EHEC occurs through the ingestion of contaminated foods such as milk, vegetable products, water-based drinks, and particularly, minced meats (Colello et al., 2016). Moreover, 5–10% of the patients infected with EHEC develop the more severe hemolytic-uremic syndrome (HUS). HUS is the most common cause of acute renal failure and the second cause of chronic renal failure and renal transplantation in children. Therefore, STEC/EHEC constitutes a serious threat to public health and a major concern for the sustainability of the meat industry as well as for its entire production chain. Presently, consumers assumed a crucial role requiring safer and healthier foods. This context highlights the need to provide the meat industry with sustainable and eco-friendly solutions to limit and prevent future risks surrounding this problematic.

Lactic acid bacteria (LAB), naturally present in meat, are of technological interest due to their inhibitory potential on spoilage, toxin production or food poisoning microorganisms in foodstuffs (Vignolo et al., 2015). Their antagonism toward spoilage bacteria is due to the direct competition for nutrients and/or production of different antimicrobial metabolites, such as organic acids, hydrogen peroxide, and bacteriocins (Woraprayote et al., 2016). In particular, by producing lactic acid and thus lowering the pH, LAB inhibit the growth of bacterial pathogens and even kill them (Atassi and Servin, 2010). Moreover, some of them produce bacteriocins, ribosomally synthesized peptides with antibacterial activity toward closely related strains, playing an important role in food preservation. Some type of LAB bacteriocins are specifically active toward Gram positive spoilage and pathogenic microorganisms such as *Listeria monocytogenes* and *Brochothrix termosphacta* (Woraprayote et al., 2016). Due to these properties, the use of LAB is an interesting substitute for chemical and/or physical preservatives. Moreover, LAB are generally regarded as safe (GRAS) and usually fit all recommendations for food usage (Wessels et al., 2004). These characteristics make LAB ideal candidates for the development of bioprotective agents, providing a good antagonistic activity toward target organisms (Chikindas et al., 2017). It is known that most of LAB bacteriocins are not effective against Gram negative microorganisms such as *E. coli*, although they can became active in association with agents such as EDTA or organic acids, affecting membrane integrity of the target organisms (Belfiore et al., 2007). Even though, no bioprotective LAB culture capable of inhibiting EHEC in meat is available on the market so far (Varsha and Nampoothiri, 2016).

On this basis, it is proposed that certain LAB could control and/or inhibit the growth of EHEC in meat through direct or indirect interaction with the pathogen. In order to proceed toward an efficient bioprotective culture as strategy of EHEC control for meat preservation, it is necessary to have a highly competitive strain to fight the pathogen. The knowledge of the mechanisms by which both microorganisms interact is therefore of paramount importance. On this context, the objective of this work was to evaluate the potential of LAB for inhibiting EHEC. The assayed LAB strains were examined for antagonistic activity toward EHEC by using a simple and rapid method and by analyzing the growth kinetics of co-cultures (LAB-EHEC) in a meat-based medium. A comparative proteomic approach was used to identify the underlying mechanisms involved in the antagonistic action carried out by the selected LAB strain.

## Materials and methods

### Bacterial strains and culture conditions

*Lactobacillus curvatus* CRL705, *Lactobacillus plantarum* CRL681 isolated from artisanal fermented sausages and *Enterococcus mundtii* CRL35 of cheese origin, belonging to CERELA culture collection were used. They were selected for this study, due to their well-studied biochemical, bioprotective activity toward *Listeria monocytogenes* and/or their technological features (Fadda et al., 1998, 1999, 2010; Saavedra et al., 2004; Salvucci et al., 2007).

Fresh cultures were obtained from freeze-dried stocks and transferred twice in MRS (Merck, Buenos Aires, Argentina) (De Man et al., 1960) incubated at 30°C for 24 h and used for further inoculation. The stock culture was stored at −80°C in milk yeast extract medium (10% w/v skim milk, 0.5% w/v yeast extract) containing 10% (v/v) glycerol as cryo-protectant.

The atoxigenic *Escherichia coli* O157:H7 NCTC12900 (National Type Culture Collection, Colindale, London) was selected as the pathogen model to evaluate LAB-EHEC interaction. *E. coli* NCTC12900 was isolated in Austria in 1992 and does not produce enterotoxins Stx1 nor Stx2 (Best et al., 2003). This strain was kept at −80°C in LB (Luria Bertani) medium in the presence of 20% (v/v) glycerol as cryo-protectant. To obtain fresh cultures, the strain was transferred twice in LB broth and incubated at 37°C for 8 h, in the first transfer, and for 16 h in the second transfer.

### *E. coli* growth inhibition assay

The inhibitory capacity of the strains was evaluated by the well-diffusion assay according to Salvucci et al. (2007) with some modifications. Briefly, 5 μl of each treatment of LAB culture were spotted onto a plate containing MRS agar. The indicator lawn was prepared by adding 100 μl of an overnight culture of EHEC to 10 ml of LB soft agar (0.7%); poured on top of MRS agar inoculated with each strain. The plates were incubated at 30°C for 24 h. In order to evaluate, the mechanisms of inhibition toward EHEC, different conditions were assayed for each LAB strain: (1) intact/viable cells, removing the effects of soluble factors from the supernatant. Cell suspensions were washed with physiological solution and spotted onto MRS agar; (2) non-viable cells, cell suspensions from the overnight culture washed with distilled water and heated 15 min at 95°C; (3) cells collected, washed with physiological solution and resuspended in 1 mg/ml lysozyme solution incubated 2 h and spotted onto MRS agar, to evaluate if cell wall is involved in *E. coli* inhibition; (4) overnight LAB culture in MRS was directly spotted onto MRS agar, to evaluate all components (viable cells plus all metabolic products) present in the medium; (5) cell–free supernatant of the overnight culture heated (5 min, 95°C) and spotted onto the MRS agar, to evaluate bacterial inhibition due to acid, bacteriocins, and other heat stable compounds; (6) cell-free supernatant of the overnight culture heated (5 min, 95°C), and neutralized to pH 7 with 1N NaOH and spotted onto MRS agar, to neutralize the acids produced; (7) untreated cell-free supernatant was spotted onto the MRS agar, to evaluate additional soluble factors that could inhibit the pathogen; (8) 4% lactic acid solution was spotted as a control of the acid effect.

### Sarcoplasmic model system

The sarcoplasmic model system was used as culture medium and prepared according to Fadda et al. (1998) with some modifications. Briefly, 10 g of bovine *semimembranosus* muscle were homogenized with 100 ml of deionized water for 8 min in a Stomacher 400 blender (Stomacher, London, UK). The homogenate was centrifuged (14,000 g, 20 min at 4°C). The supernatant containing sarcoplasmic proteins was filtered through Whatman paper, filter-sterilized through a 0.22 μm-pore-size filter (Steritop GP, Biopore, Buenos Aires, Argentina) and supplemented with 0.5% (w/v) glucose and 0.01% (v/v) Tween 80. The sterility of the system was confirmed by plating in Plate Count Agar (PCA).

### LAB—EHEC co-cultures in sarcoplasmic model system. focus on LAB inhibitory potential

Co-cultures of each LAB strain with *E. coli* NTCC12900 were carried out in the sarcoplasmic model to evaluate the performance of both microorganisms in co- and individual culture. Fifty ml of the sarcoplasmic model was inoculated with 10^6^ CFU/ml of LAB and 10^4^ CFU/ml of *E. coli* and incubated under gentle stirring at 30°C for 96 h. In addition, each microorganism was grown individually under the same conditions (30°C, 96 h; same inoculum) to evaluate the behavior of each strain without competition.

Samples were taken at 0, 3, 6, 8, 24, 48, 72, and 96 h to analyze pH and viability of both microbial groups using selective agar media. For bacterial enumeration, decimal dilutions were prepared and plated on the corresponding medium, MRS agar for LAB and Mac Conkey agar (Britania, Buenos Aires, Argentina) for *E. coli*, and incubated at 30°C for 48 and 24 h, respectively. Measurements of pH were determined by using pHmeter Altronix TPX I (New York, USA).

Three independent cultures were carried out for each mixed and independent cultures.

### Proteomic study

For differential proteomic analysis, one strain, *Ent. mundtii* CRL35, was selected to evaluate LAB – *E. coli* interaction in the sarcoplasmic model system by means of two dimensional electrophoresis (2DE). Two different time periods during the growth were evaluated: T6, corresponding to 6 h of growth when both microorganisms (LAB and *E. coli* NTCC 12900) were in the exponential phase of growth, both in single or co-cultures, and T30 corresponding to 30 h when the stationary phase was achieved by LAB (in both single and co-culture), while *E. coli* in co-culture was already in the death phase. However, when *E. coli* grew alone 96 h was taken as the second sampling time, this corresponding to the death phase for the pure culture of *E. coli*.

#### Cells recovery for proteomic analyses

*Ent. mundtii* CRL35 was incubated as individual and co-culture with *E. coli* O157:H7 NTCC12900, as described before, in 100 ml of the sarcoplasmic model system to achieve sufficient amount of cells for proteomic analysis. Cells from co-cultures were harvested at 6 h (T6) and 30 h (T30). Cells from single cultures of *Ent. mundtii* were also harvested at 6 h (T6) and 30 h (T30). As mentioned before, cells from *E. coli* growing alone were collected at 6 h (T6) and 96 h (T96). Cells from different cultures were harvested by centrifugation at 8,000 × g for 10 min at 20°C and twice washed with 40 ml 0.1M Tris-HCl buffer, pH 7.5 and centrifuged at 8,000 × g for 10 min at 20°C. The resulting pellets were stored at −20°C until lysis for protein extraction. Three independent biological replicates were performed for each condition.

#### Preparation of cell free protein extracts for proteomic analyses

**Figure 1:**
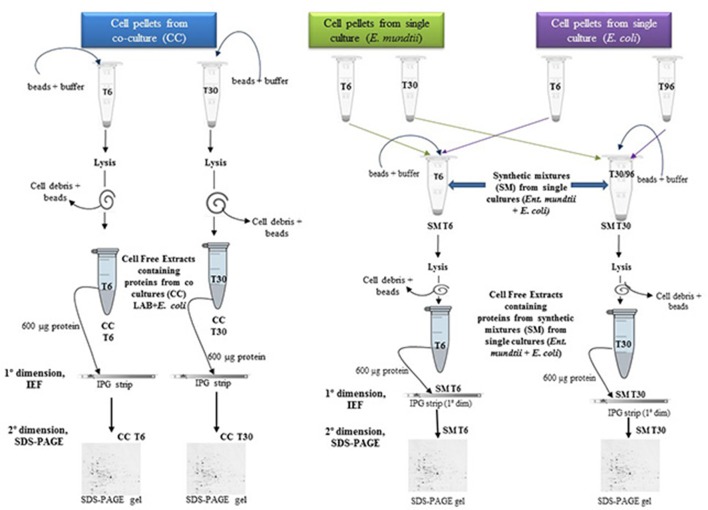
Schematic description of cell free extracts preparation for proteomic analysis.

The cell pellets from the co-cultures were mixed with glass beads (150 ± 212 μm diameter, Sigma-Aldrich Co., St. Louis, MO, USA) and further re-suspended in 0.1 M Tris-HCl buffer, pH 7.5 in a 1:2:1 (cell:buffer:bead) ratio. Then, cells were disrupted using a Mini-BeadBeater-8 cell disrupter (Biospec Products Inc., Bartlesville, OK, USA) at maximum speed for 10 min (10 cycles of 1 min each, with 1-min intervals on ice among cycles). To remove cell debris, unbroken cells and glass beads, samples were centrifuged (14,500 × g, 5 min, 15°C). The supernatant constituted the cell free extract. The protein concentration of this extract was estimated according to Bradford assay using bovine serum albumin as a standard. The whole process is described in Figure 1. Aliquots of 600 μg of protein were finally stored at −80°C, until further analysis.

*Ent. mundtii* and *E. coli* cell pellets from individual cultures at T6 were mixed before lysis and the same was performed for T30/T96 (see schematics in Figure 1). This procedure was carried out to standardize and avoid differences in cell lysis efficiency between mono and co-cultures, as well as problems with differences in protein enrichment of each microorganism in 2DE gels, that could affect proteome comparisons. The cell proportions of each microorganism used for T6 and T30 mixtures coming from single cultures were established according to the cell counts obtained in the respective co-culture in order to have in the mix a similar ratio between the two microorganisms. This way, assuring to have 600 μg of proteins placed in the IPG strip, with the same protein proportion of each microorganism than in IPG strips with samples from the co-culture. Each of these synthetic mixtures (SM) will constitute the respective controls (single culture) at T6 and T30 respectively. The SM were mixed with glass beads and subjected to lysis as previously described for cell pellets originating from co-cultures (see Figure 1 for details).

#### Two-dimensional gel electrophoresis

Sample preparation and 2DE gels were carried out according to Bustos et al. (2015). Isoelectrofocusing (IEF) was performed in IPGphor (GE Healthcare, Uppsala, Sweden) at 53,500 Vh, using the immobilized pH gradient (IPG) strips (Immobiline DryStrip Gels, linear pH 4–7, 18 cm, GE Healthcare; Uppsala, Sweden). For the second dimension, IEF strips were equilibrated at room temperature in 6 M urea, 2% (w/v) SDS, 30% (w/v) glycerol, 50 mMTris-HCl, pH 8.0, containing alternatively 50 mM DTT (15 min) and then 400 mM iodoacetamide (15 min in the dark). Second dimension was performed on homogeneous 12.5% (w/v) polyacrylamide gels at the constant current of 15 mA/gel at 15°C (~16 h) using an Ettan DALTsix Large Vertical System (GE Healthcare, Uppsala, Sweden). Gels were stained with colloidal Coomassie blue Stain according to Candiano et al. (2004), distained with distilled water. The 2DE maps were digitalized using Image Scanner III LabScan 6.0 (GE Healthcare, Uppsala, Sweden).

#### Image acquisition and data analysis

Volume spot quantization and normalization were performed on digitalized gel images (600 dpi) using the software Prodigy SameSpots version 1.0.3400.25570 (Totallab, Newcastle, UK). The volume of each spot was calculated and normalized by referring the values to the sum of total spot volumes within each gel. Student test for unpaired samples was applied. A protein was considered differentially abundant if the mean normalized spot volume varied at least 1.5-fold between compared spots. The effect was confirmed by analysis of variance at a significance level of *p* < 0.05. Protein spots showing significant variation between studied conditions were manually excised from the gels using a scalpel blade and identified using Mass Spectrometry.

#### Mass spectrometry protein identification

Selected spots were excised from the corresponding gel, digested with trypsin and, submitted to tryptic digestion and then to mass spectrometry analyses as previously described (Nally et al., 2017). Tryptic peptides were subsequently ionized using α-cyano-4-hydroxycinnamic acid as matrix. Mass spectrometric analysis of the peptide solutions was carried out on a MALDI-TOF/TOF tandem mass spectrometer ABI 4700 proteomics analyzer (Applied Biosystems, Foster City, USA) according to Grosu-Tudor et al. (2016) or on a MALDI 5800 (Sciex, Foster City, USA) and performed at CEQUIBIEM (Facultad de Ciencias Exactas y Naturales, UBA, Buenos Aires, Argentina) and at LIST - Luxembourg Institute of Science and Technology “Environmental Research and Innovation” (ERIN), respectively. MASCOT search engine (Matrix Science Inc., Boston, MA; http://www.matrixscience.com/search_form_select.html) was used to identify proteins from peptide mass fingerprint data based on the annotated genome of *Ent. mundtii* CRL35 (https://www.ncbi.nlm.nih.gov/nuccore/JDFT00000000). All proteins were identified using BLASTp in NCBI database (Altschul et al., 1990).

### Functional analysis and interaction of proteins

The functional study of identified proteins and their classification into functional categories were performed using the databases Universal Protein Resource (UniProt) (UniProt Consortium, 2015) and COGNITOR to identify the Clusters of Orthologous Groups of proteins (COGs) (Galperin et al., 2015).

To explore the interactions between the proteins that have shown differential expression, we conducted an *in-silico* analysis using the publicly available STRING version 10.05 (database Search Tool for the Retrieval of Interacting Genes/Proteins) (Szklarczyk et al., 2015). For the differentially over expressed proteins, the number of protein-protein interactions documented in the database were determined. For visualization purposes, a graph was constructed linking proteins represented by nodes with known interactions with the identified proteins. All available prediction methods on STRING were used and 0.4 was select as confidence level (Szklarczyk et al., 2015).

### Statistical analyses

All experiments (growth inhibition, growth kinetics and differential protein expression assays) were done three times, and the values and the standard error were calculated from the data with three repetitions. One-way analysis of variance with *t*-test was conducted, and a *p* value of less than 0.05 was considered to indicate a statistically significant difference. The hypergeometric distribution was assayed to evaluate the enrichment of COG categories of the proteins encoded by *Ent. mundtii* CRL35 related to the ones differentially expressed by it in co-culture or alone (CC T30 - SM T30) and in co-culture during the time (CC T6 – CC T30).

## Results

### *E. coli* growth inhibition assay

**Figure 2:**
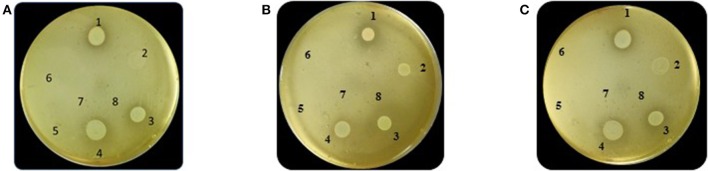
Results of the Plate inhibition assay of LAB toward EHEC. Studied conditions of *Ent. mundtii* **(A)**, *L. plantarum* CRL 681 **(B)** and *L. curvatus* CRL 705 **(C)** toward *E. coli* NCTC12900: 1- LAB cell suspension in physiological solution, 2- LAB cell suspension in distilled water and heated 15 min at 95°C, 3- LAB cells in 1 mg/ml lysozyme solution, 4- direct overnight LAB culture in MRS, 5- heated supernatant (5 min, 95°C), 6- heated (5 min, 95°C), and neutralized supernatant, 7- intact supernatant, 8- 4% lactic acid.

The *E.coli* growth inhibition assay was performed as a first approach to determine the antagonistic potential of LAB strains toward the pathogen (Figures 2A–C). By means of this fast and simple method the inhibitory capacity of each LAB strain on *E. coli* NTCC12900, as well as the nature of the inhibition effect were evaluated. The three assayed LAB showed a similar inhibitory pattern, although they presented low variability in their inhibition halos. In general, halos of inhibition were registered in those conditions where LAB cells remained viable and not seriously damaged; regardless of the presence of the culture supernatant (spot #1 and 4). When cells were treated with lysozyme, minor inhibition halos were present (spot #3). In contrast, no inhibition were observed when lysed cells or cell free supernatant (untreated, heated or heated and neutralized) containing metabolites such as organic acids, heat stable bacteriocins and different soluble factors were spotted (spot #5, 6 and 7). The absence of inhibitory halos when 4% lactic acid solution was spotted (spot #8) confirmed the known acid resistance of *E. coli* (Figure 2).

### Performance of LAB and *E. coli* NCTC 12900 when grown individually or in co-culture in sarcoplasmic model system

**Figure 3:**
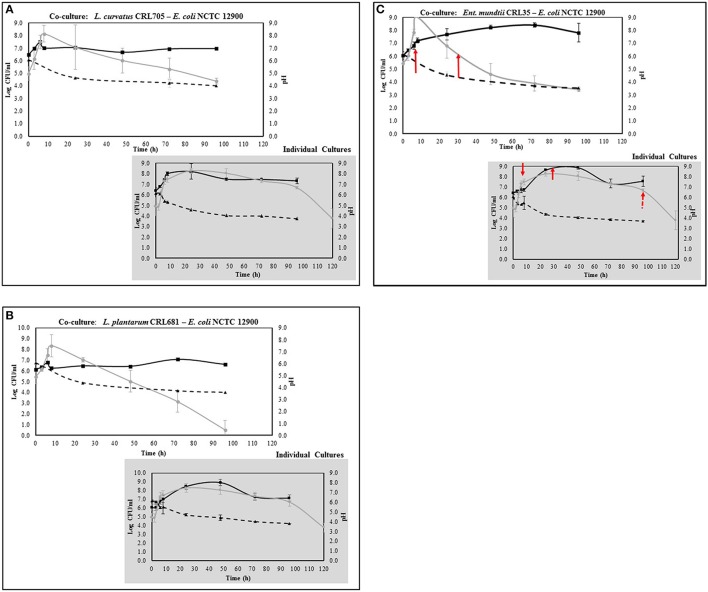
Kinetics of LAB (black line) and *E. coli* (gray line) growth (Log CFU/ml) in co- and individual culture in the sarcoplasmic model system at 30°C. The pH is represented with dashed line. **(A)** Co-culture and individual culture of *L. curvatus* CRL705, **(B)** Co-culture and individual culture of *L. plantarum* CRL681, **(C)** Co-culture and individual culture of *Ent. mundtii* CRL35. The red arrows indicate the time of sampling for proteomic assays.

When grown individually, LAB presented an adequate growth in the sarcoplasmic model system, achieving a maximal growth approximately at 24–48 h depending on the strain (2.0 × 10^8^ – 9.8 × 10^8^ CFU/ml) (Figures 3A–C). They reached the exponential growth between 3 and 8 h and the stationary growth phase around 24 h. Afterwards, LAB strains maintained approximately initial bacterial counts until 96 h of incubation (1 × 10^6^ CFU/ml). On the other hand, a significant pH drop was observed in all LAB strains growing alone. When *E. coli* was inoculated alone, it also achieved an optimal growth presenting a traditional sigmoid kinetic curve, the exponential growth was attained between 4 and 6 h with the maximal cell viability at 24 h during the stationary phase (1.8 × 10^8^ CFU/ml) (Figures 3A–C). When LAB-*E. coli* co-cultures were analyzed, a different growth kinetic was achieved by both type of microorganisms compared to its individual growth. A decreased growth rate of LAB in the presence of *E. coli* was observed, achieving 1–2 logarithmic units less of cell viability than in the single culture condition, depending on the strain. However during co-culturing, all LAB were able to keep the steady state until the end, with a population almost similar to the beginning. It is worth noting that the acidifying potential of LAB was not affected by the presence of the pathogen, reaching similar values than those observed in pure cultures (final pH between 4.5 and 3.7) (Figures 3A–C). On the other hand, the growth of *E. coli* in co-culture was affected considerably by LAB, specifically after the first 8 h. For instance *L. curvatus* CRL705 (bacteriocin producer), showed a slight decrease of *E. coli* population, ~0.6 log units (Figure 3A). Whereas the bacteriocinogenic strain *Ent. mundtii* CRL 35 (Figure 3C) and *L. plantarum* CRL 681 (non-bacteriocin producer) (Figure 3B), showed higher inhibitory effect. In fact, their presence produced a significant reduction of *E. coli* viability after 8 h of growth, accelerating the entrance of *E. coli* into the death phase. At 96 h a significant decrease of *E. coli* counts was observed reaching more than 2 and 3.5 logarithmic units of decrease in the co-cultures containing *Ent. mundtii* CRL35 and *L. plantarum* CRL681, respectively (Figures 3B,C).

### Differential protein expression of *Ent. mundtii* CRL35 in co-culture with *E. coli* NCTC12900

*L. plantarum* CRL681 and *Ent. mundtii* CRL35 resulted the most effective strains to fight against *E. coli* in co-cultures. However, we decided to select *Ent. mundti* CRL35 for the proteomic analyses due to the availability of its genome, partially sequenced and annotated, which is essential for protein identification during proteomic studies. Additionally, this strain is able to produce the Enterocin CRL35, a very effective bacteriocin toward *Listeria monocytogenes*. The well-established bioprotective activity of *Ent. mundtii* CRL35, offers an extended range of bioprotection, not only against *E. coli* O157:H7 but also against other food-borne pathogens of concern to meat industry (Salvucci et al., 2007). Finally, *Ent. mundtii* CRL35 possess complete biochemical and genetic studies that guarantee its technological and bioprotective features that make this strain an interesting candidate to be used as adjunct culture in food (Saavedra et al., 2004; Pingitore et al., 2012). Nevertheless interaction studies between *E. coli* and *L. plantarum* CRL681, as well as, the effect of both LAB toward *E. coli* O157:H7 are focus of future experiments.

**Figure 4:**
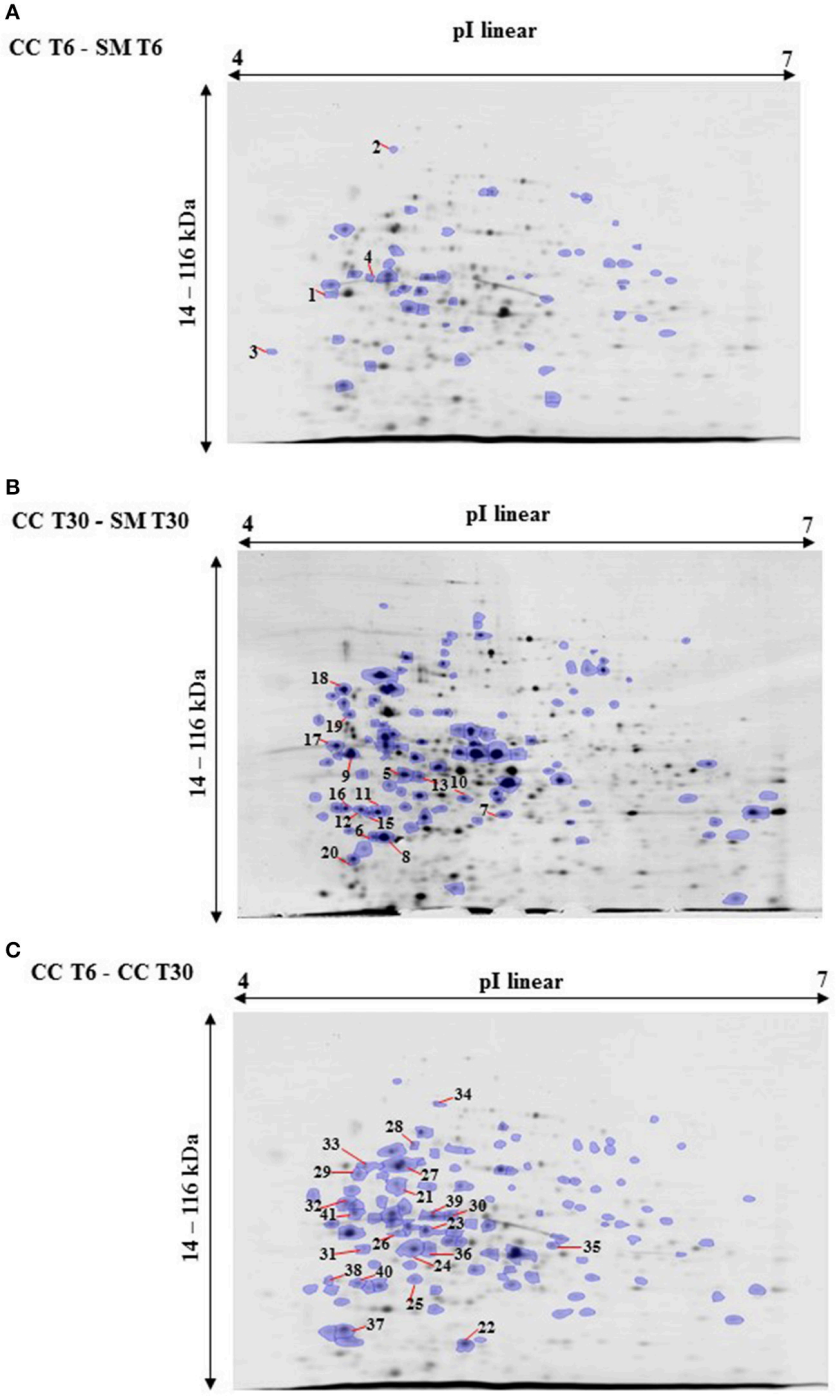
2DE gels showing the proteins differentially expressed of both microorganisms. The differentially expressed and successfully identified proteins of *Ent. mundtii* are numbered. **(A)** Co-culture T6 vs. individual growth T6 (CC T6 - SM T6). **(B)** Co-culture T30 vs. individual growth T30 (CC T30 - SM T30). **(C)** Co-culture T6 vs. co-culture T30 (CC T6 - CC T30).

In order to study the interaction between *Ent. mundtii* CRL35 and *E. coli* O157:H7 NCTC12900 and to identify the molecular mechanisms underlying the antagonistic action carried out by *Ent. mundtii* in a meat environment, differential protein expression during the growth of *Ent. mundtii* alone or in co-culture was evaluated by 2DE. Two key moments of the growth were analyzed: 6 h (T6), when both microorganisms grew exponentially, and 30 h (T30), when *Ent. mundtii* achieved the stationary phase and *E. coli* entered in death state during co-culture (Figure 3C). Differential protein expression was evaluated according to the following comparisons: (i) *Ent. mundtii* growing in co-culture vs. *Ent. mundtii* growing individually at T6 (CC T6 - SM T6); (ii) *Ent. mundtii* growing in co-culture vs. *Ent. mundtii* growing individually at T30 (CC T30 - SM T30) and iii) *Ent. mundtii* growing in co-culture at T6 vs. *Ent. mundtii* growing in co-culture at T30 (CC T6 - CC T30). Representative 2DE maps of the bacterial proteomes when grown alone or in co-cultures are depicted in Figure 4.

In the three proteomic analyses, the most significant differentially expressed proteins (*p* < 0.05, fold > 1.5), 106 spots, were submitted to MS identification. Of these, a total of 91 proteins were successfully identified, 50 belonged to *E. coli* and 41 to *Ent. mundtii*, according with protein databases. In the present work, we focused in proteins related to *Ent. mundtii* CRL35 proteome (Tables 1, 2).

**Table 1:** Identified overexpressed proteins during *Enterococcus mundtii* CRL35 growth in co-culture respect to its individual growth at T6 (6 h) and T30 (30 h) in the sarcoplasmic model system at 30°C.

**Function**	**COG^a^**	**Spot^b^**	**Protein name**	**Accession N°^c^**	**Score^d^**	**Gene**	**Theoretical MM^e^**	**pI^f^**	**Overexp/Fold change^g^**	**Detailed 2DE gels showing differentially expressed spot in: Co-culture/Individ.growth**
**T6**										
Carbohydrate Metabolism	G	1	Enolase	gi|602619948	1120	eno	46496	4.60	1.9	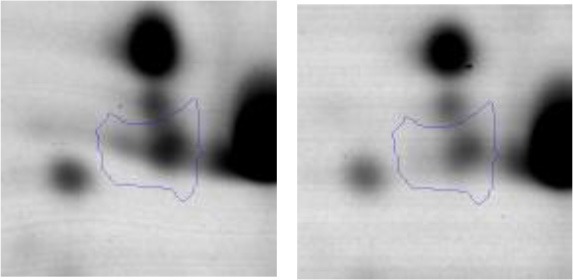
Amino acid metabolism	E F	2	Carbamoyl Phosphate Synthase Large Subunit	gi|602619378	1170	AK89_04630	117592	4.82	1.8	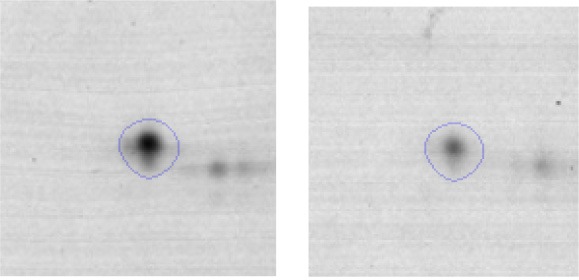
	P	3	Methionine ABC Transporter Substrate-Binding Protein	gi|602620029	668	AK89_00090	30407	4.36	2.1	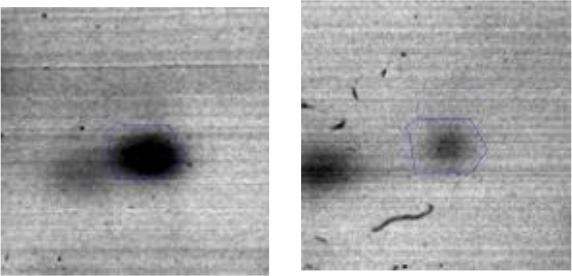
Cell division	D	4	Cell Division Protein FtsZ	gi|602618182	367	ftsZ	44517	4.73	2.2	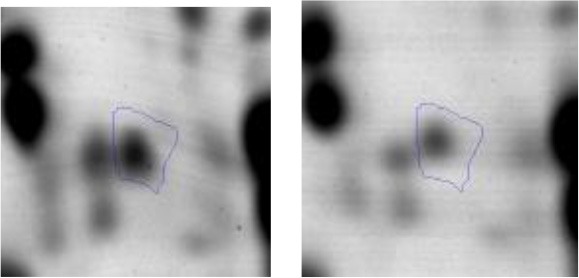
**T30**										
Carbohydrate Metabolism	G	5	Phosphoglycerate kinase	gi|602619950	1040	Pgk	42045	4.94	2.1	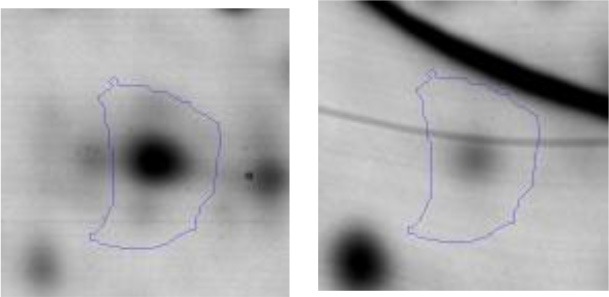
	G	6	Fructose-bisphosphate aldolase	gi|602618838	707	AK89_07810	30955	4.77	4.0	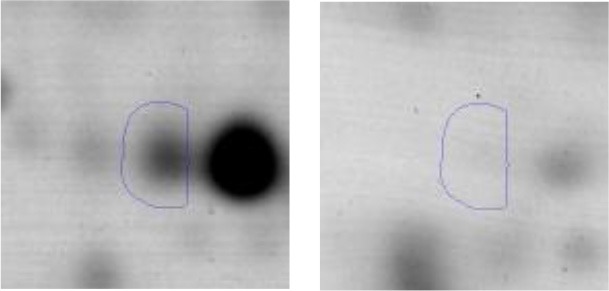
	G	7	6-phosphofructokinase	gi|602619230	1320	pfkA	34342	5.32	4.7	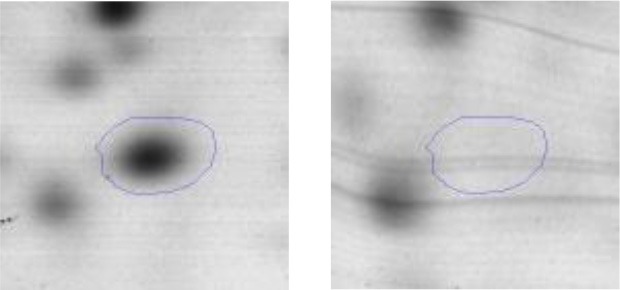
	G	8	Fructose-bisphosphate aldolase	gi|602618838	626	AK89_07810	30955	4.77	4.6	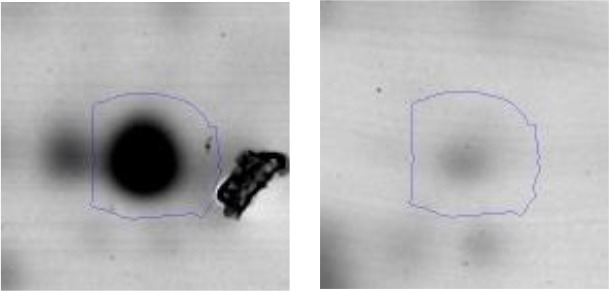
	G	9	Enolase	gi|602619948	1460	Eno	46496	4.60	2.0	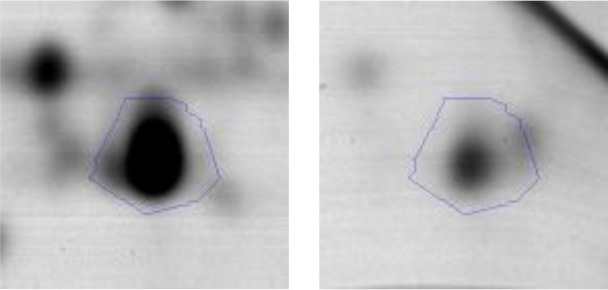
Energy production and conversion	C	10	Pyruvate dehydrogenase E1 subunitalpha	gi|602618669	938	AK89_08920	41004	5.14	2.6	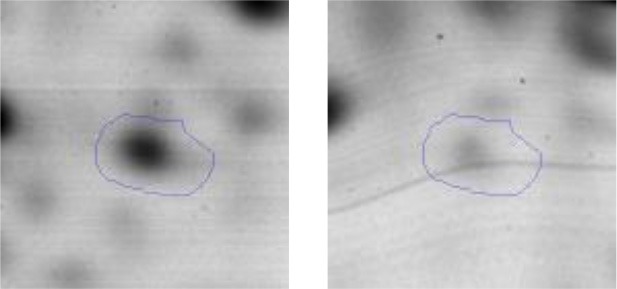
	C	11	L-lactate dehydrogenase	gi|602619124	1230	Ldh	35809	4.74	3.1	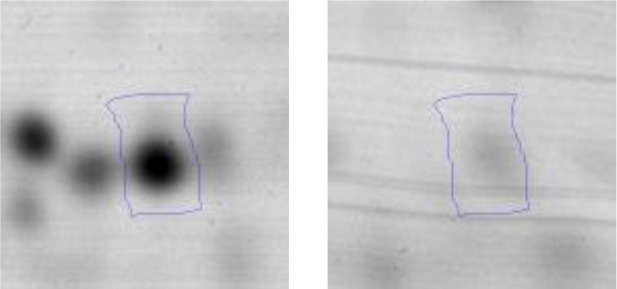
	C	12	2-oxoisovalerate dehydrogenase subunit beta	gi|602618668	1240	AK89_08915	35393	4.70	2.7	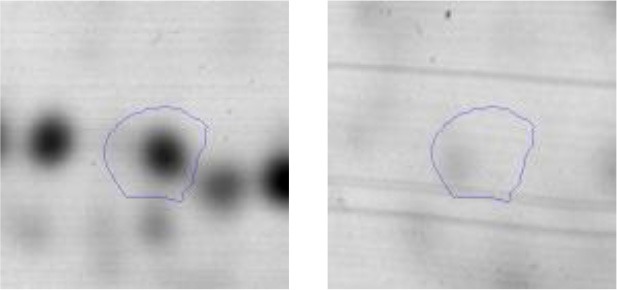
Transcription	K	13	DNA-directed RNA polymerase subunit alpha	gi|602618530	445	rpoA	35276	4.98	2.3	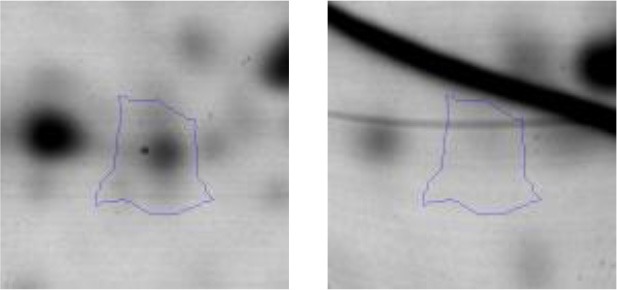
Cell division	D	14	Cell division protein DivIVA	gi|602618177	549	AK89_10945	26893	4.61	4.0	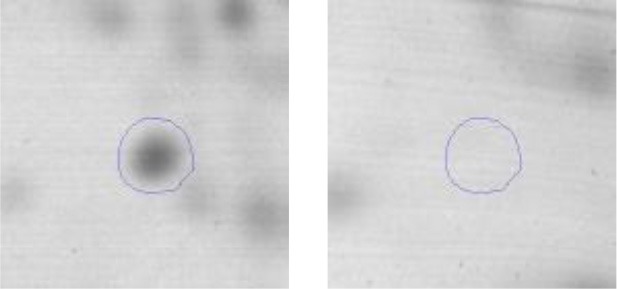
Metabolism	IQR	15	Oxidoreductase	gi|602619024	1020	AK89_06445	31882	4.77	3,5	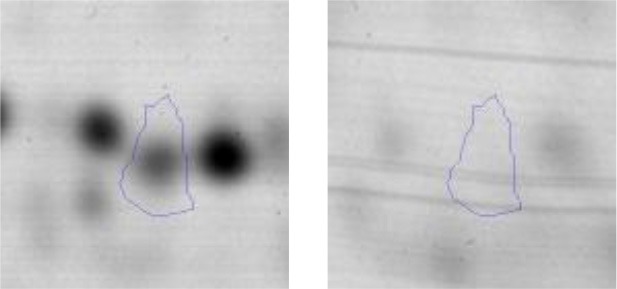
Cell wall biosynthesis	M	16	Choloylglycine hydrolase	gi|602619043	951	AK89_06560	36948	4.62	3.2	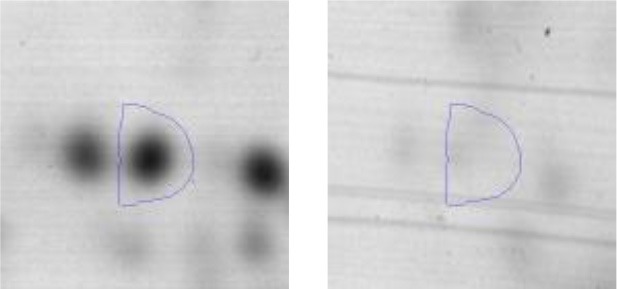
Amino acid metabolism	E	17	Hypothetical protein AK89_04275	gi|602619580	938	AK89_04275	49541	4.58	2.6	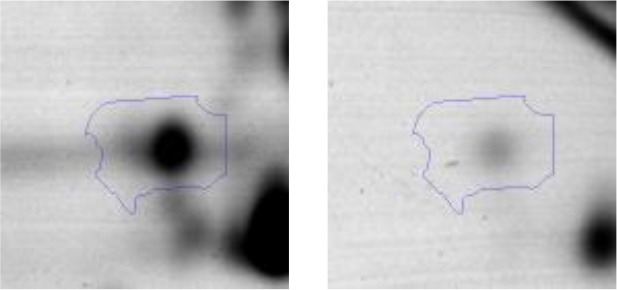
Folding and protein processing	O	18	Molecular chaperone DnaK	gi|602618370	1360	dnaK	65585	4.63	2.5	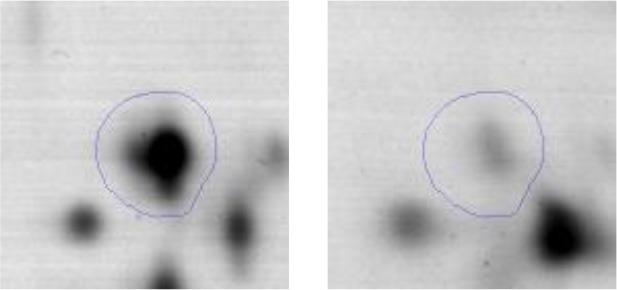
Ribosomal structure	J	19	30S ribosomalprotein S1	gi|602619573	929	AK89_04240	44564	4.66	2.5	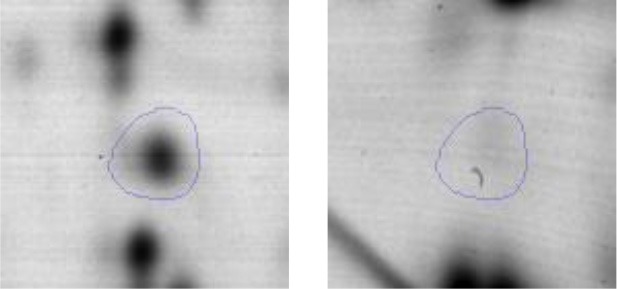
Stress	S	20	Stress response regulator Gls24	gi|602619010	873	AK89_06350	20148	4.63	2.3	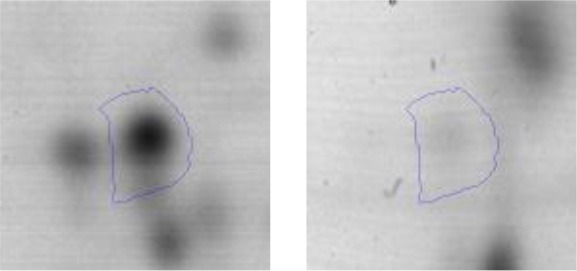

a Functional category according to COG database. One letter abbreviations for the COG functional categories: G, carbohydrate metabolism and transport; C, energy production and conversion; K, transcription; D, cell division and chromosome partitioning; I, lipid metabolism; Q, secondary metabolites biosynthesis, transport, and catabolism; R, general functional prediction only; M, cell wall structure and biogenesis and outer membrane; E, amino acid transport and metabolism; O, molecular chaperones and related functions; J, translation, including ribosome structure and biogenesis; S, no functional prediction.

b *Spot designations correspond to those of the gels shown in Figure 4*.

c Accession number in the NCBI database.

d Protein Score is −10*Log(P), where P is the probability that the observed match is a random event. Protein scores larger than 81 are considered significant (P < 0.05).

e *Molecular Mass (Da)*.

f *Calculated isoelectric point*.

g *Relative Fold change: Normalized Volumes of protein spot in co-culture/Normalized Volumes of protein spots in individual growth*.

**Table 2:** Identified overexpressed proteins during *Enterococcus mundtii* CRL35 growth in co-culture at T6 (6 h) respect to their growth in co-culture at T30 (30 h) in the sarcoplasmic model system at 30 °C.

**Function**	**COG^a^**	**Spot^b^**	**Protein name**	**Accession N°^c^**	**Score^d^**	**Gene**	**TheoreticalMM^e^**	**pI^f^**	**Overexp./Fold change^g^**	**Detailed 2DE gels showing the differentially expressed spot in: Co-cultureT6/Co-cultureT30**
Carbohydrate Metabolism	G	21	Phosphoglucomutase/phosphomannomutase	gi|498429168	108	UAC_01121	63509	4.96	−2.2	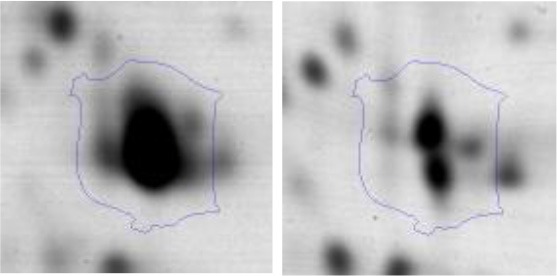
	G	22	Phosphoglycerate mutase 1 family	gi|498428689	84	gpmA	33999	6.35	2.0	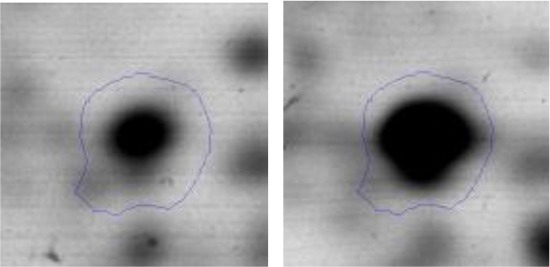
	G	23	Glucose-6-phosphate isomerase	gi|736681785	138	pgi	49574	4.99	1.9	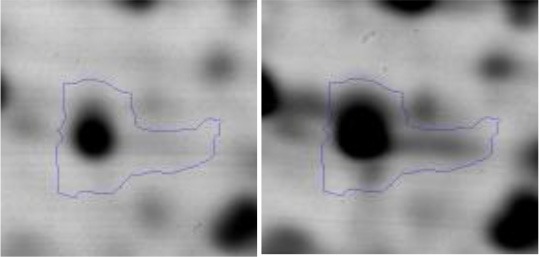
	G	24	Phosphoglyceratekinase	gi|736681519	186	pgk	42045	4.94	1.9	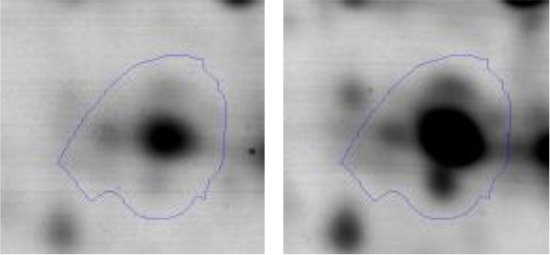
	C	25	Phosphopyruvate hydratase	gi|498429079	134	eno	46496	4.60	1.9	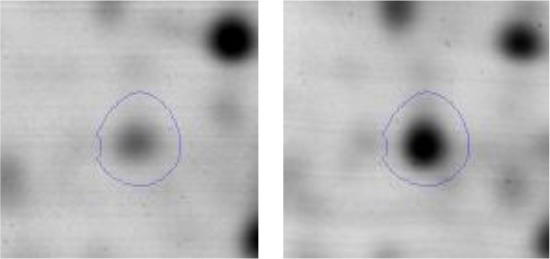
	G	26	Phosphogluconate dehydrogenase (NADP(+)-dependent, decarboxylating)	gi|498430058	134	AK89_05050	52733	4.81	2.0	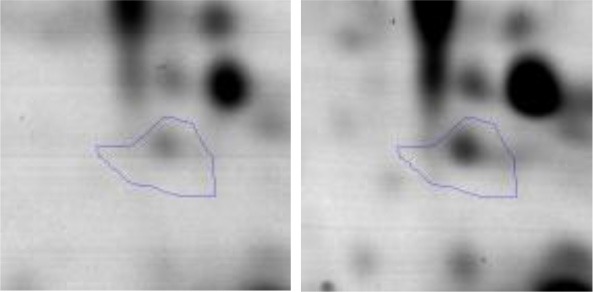
	G	27	Type I glyceraldehyde-3-phosphate dehydrogenase	gi|736681521	270	AX758_08405	35827	5.08	1.5	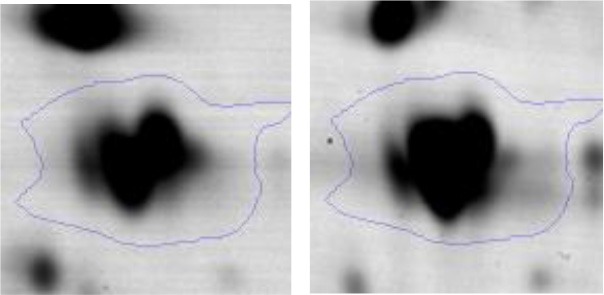
	G	28	Transketolase	gi|602617512	147	AK89_14370	49060	5.04	2.4	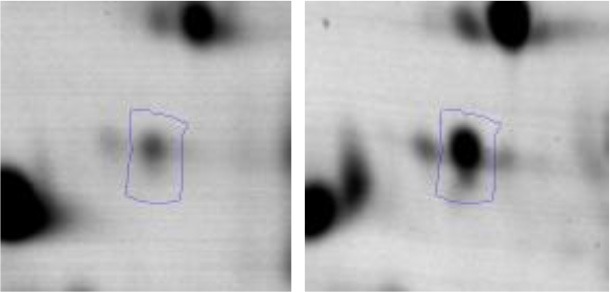
Sugar Transport	G	29	Phosphoenolpyruvate–protein phosphotransferase	gi|736682554	121	AK89_05300	63384	4.69	2.0	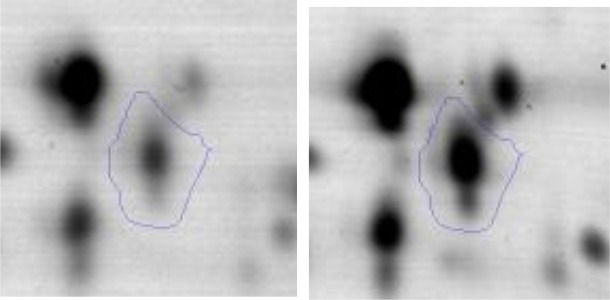
Amino acid metabolism	E	30	Glutamine synthetase	gi|498430289	109	AK89_07820	50870	5.06	2.4	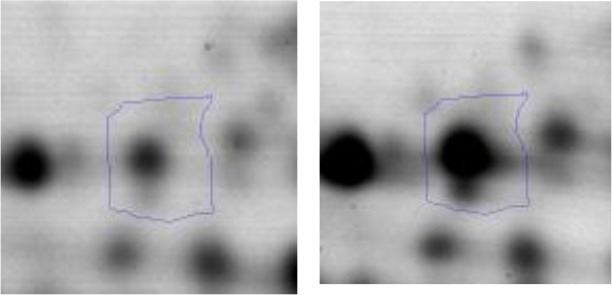
	E	31	Aminopeptidase	gi|558689945	227	pepT	45091	4.58	1.9	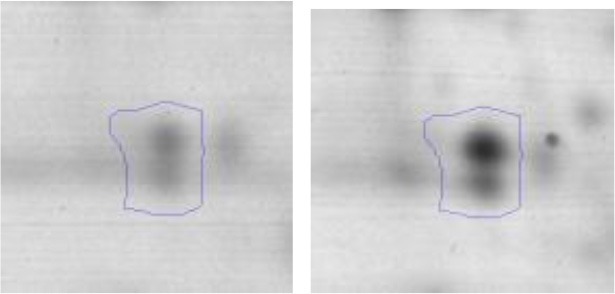
	E	32	DipeptidasePepV	gi|736683314	154	AK89_08025	51939	4.62	2.0	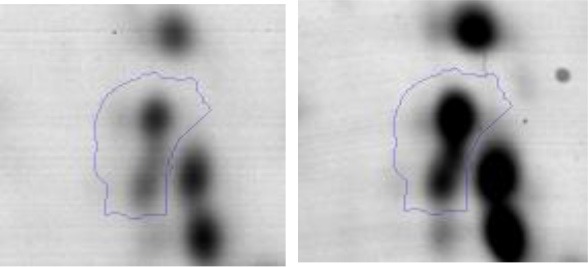
	O	33	Peptidase M13	gi|498430418	111	AK89_07270	72189	4.71	2.9	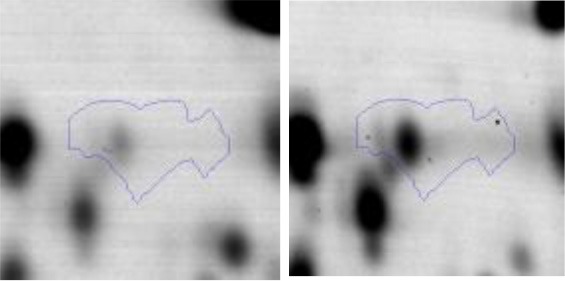
Translation	J	34	Leucine–tRNA ligase	gi|736683966	130	leuS	92016	4.99	3.4	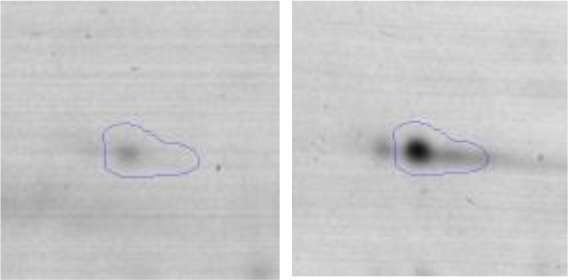
Nucleotides metabolism	F	35	Adenylosuccinate Synthase	gi|736683880	168	purA	48097	5.49	2.7	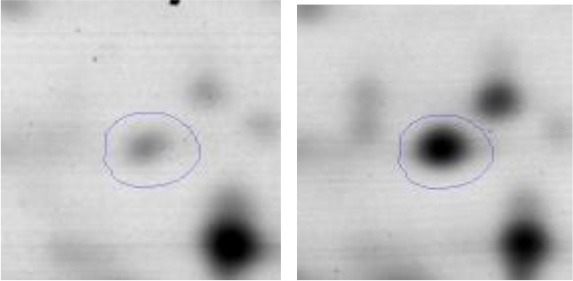
Transcription	K	36	DNA-directed RNA polymerase subunit alpha	gi|492544374	83	rpoA	35276	4.99	1.9	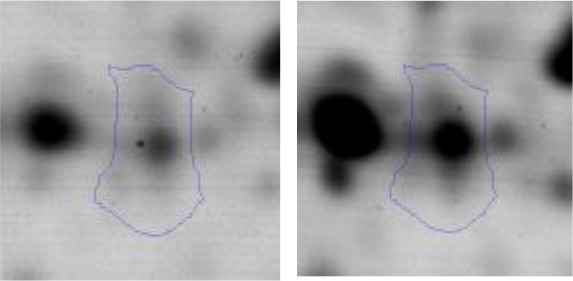
Stress	S	37	General stress protein	gi|498429607	98	AK89_02030	20130	9.19	3.1	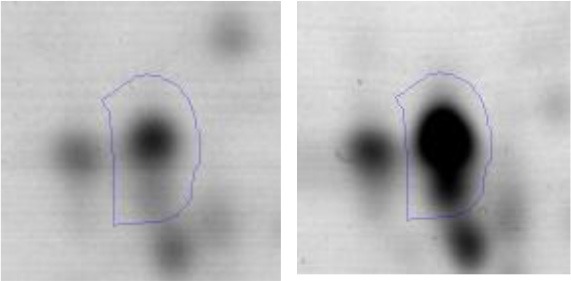
Cell wall biosynthesis	M	38	Choloylglycine hydrolase	gi|736682868	111	AK89_06560	36948	4.62	3.2	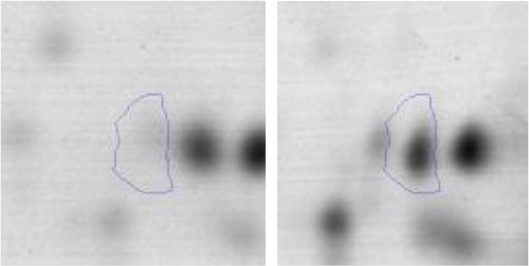
Energy production and conversion	C	39	ATP synthase subunit alpha	gi|63091861	106	atpA	56590	5.01	2.0	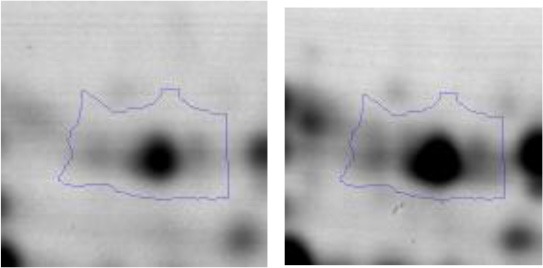
	C	40	2-oxoisovalerate dehydrogenase	gi|736683518	243	AK89_08915	35393	4.70	1.9	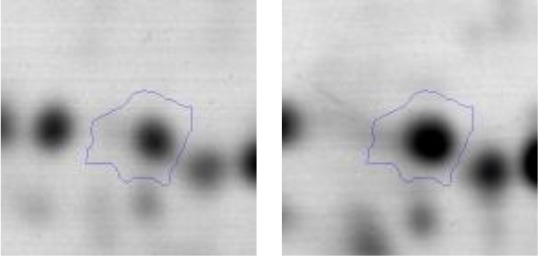
	C	41	F0F1 ATP synthase subunit beta	gi|736681478	84	atpD	51170	4.72	2.0	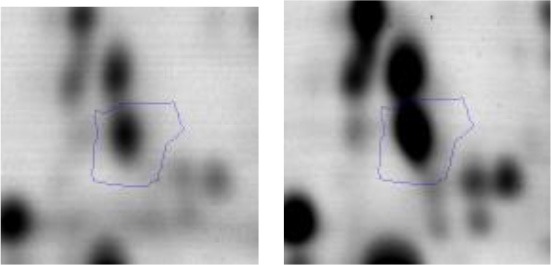

a Functional category according to COG database. One letter abbreviations for the COG functional categories: G, carbohydrate metabolism and transport; C, energy production and conversion; K, transcription; D, cell division and chromosome partitioning; I, lipid metabolism; Q, secondary metabolites biosynthesis, transport, and catabolism; R, general functional prediction only; M, cell wall structure and biogenesis and outer membrane; E, amino acid transport and metabolism; O, molecular chaperones and related functions; J, translation, including ribosome structure and biogenesis; S, no functional prediction.

b *Spot designations correspond to those of the gels shown in Figure 4*.

c *Accession number in the NCBI database*.

d Protein Score is −10*Log(P), where P is the probability that the observed match is a random event. Protein scores larger than either 81 are considered significant (P < 0.05).

e *Molecular Mass (Da)*.

f *Calculated isoelectric point*.

g *Relative Fold change: Normalized Volumes of protein spot in co-culture/Normalized Volumes of protein spots in individual growth*.

#### Differential protein expression of *Ent. mundtii* CRL35 growing alone or in co-culture

**Figure 5:**
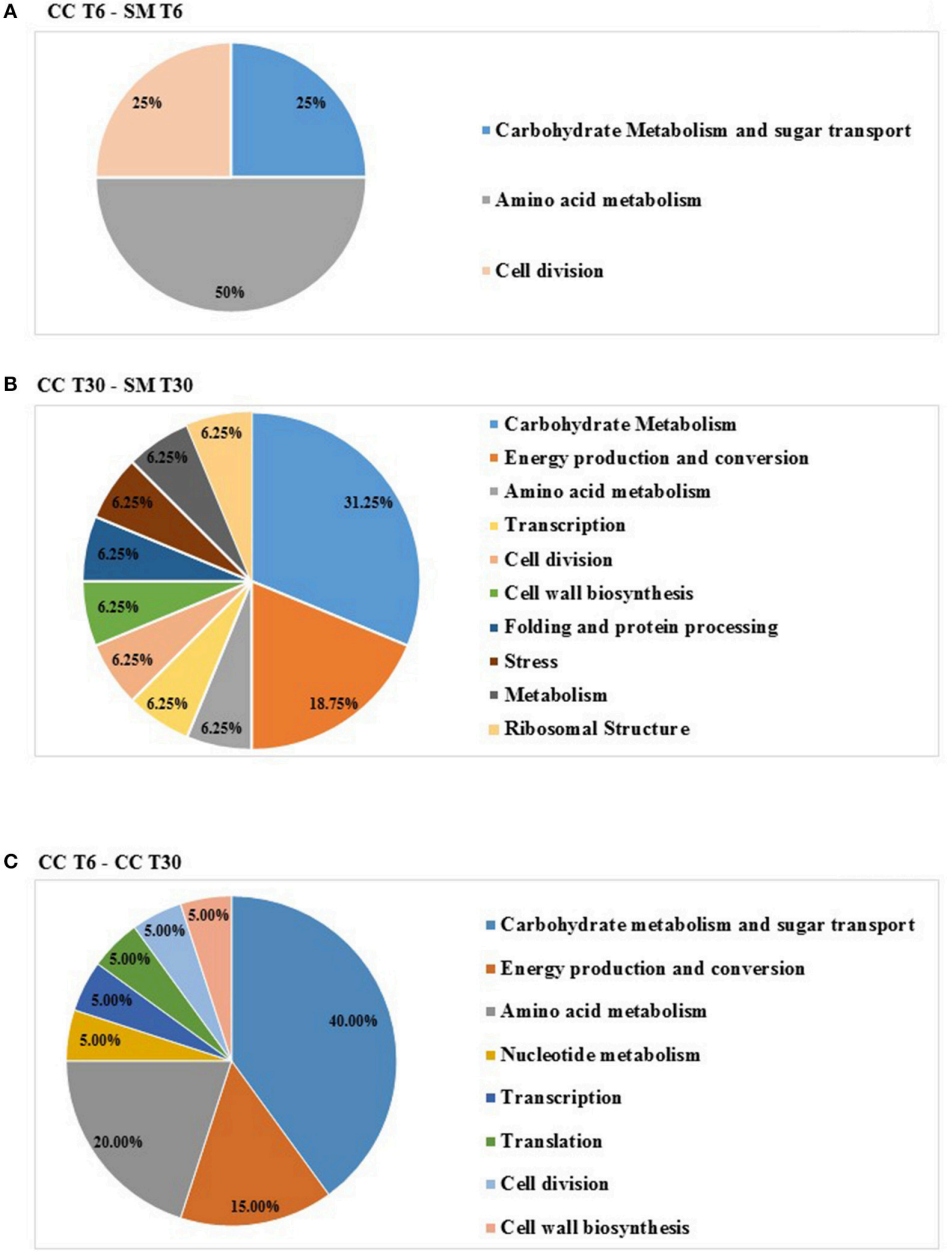
Relative abundance (%) of *Ent. mundtii* identified proteins, grouped according to their functional category, differentially expressed in: **(A)** Co-culture 6 h vs. individual growth 6 h (CC T6 - SM T6); **(B)** Co-culture 30 h vs. individual growth 30 h (CC T30 - SM T30; **(C)** Co-culture 6 h vs. Co-culture 30 h (CC T6 - CC T30).

When proteomes from *Ent. mundtii* CRL35 grown in co-culture were compared with *Ent. mundtii* growing alone in the sarcoplasmic model system, a significant protein over expression in co-culture was obtained at both analyzed times (T6 and T30). The identified proteins were successfully assigned to different functional categories. Specifically, at the first 6 h, 4 proteins related to carbohydrate (spot #1, enolase) (25%), amino acid metabolism (carbamoyl phosphate synthase large subunit, methionine ABC transporter substrate-binding Protein) (50%) and cell division (cell division protein FtsZ) (25%), resulted significantly over expressed by *Ent. mundtii* in co-culture (Table 1, Figure 5). On the other hand, after 30 h of growth in co-culture, 16 identified proteins resulted over expressed with a significant difference between 2.1 and 4.7 fold change. These proteins were involved in carbohydrate metabolism (phosphoglycerate kinase, fructose-bisphosphatealdolase, 6-phosphofructokinase, fructose-bisphosphatealdolase and enolase) (31.25%), energy production and conversion (pyruvate dehydrogenase E1 subunit alpha, L-lactate dehydrogenase, 2-oxoisovalerate dehydrogenase subunit beta) (18.75%), transcription (DNA-directed RNA polymerase subunit alpha) (6.25%), cell division (Cell division protein DivIVA oxidoreductase) (6.25%), cell wall biosynthesis (choloylglycine hydrolase) (6.25%), amino acid metabolism (hypothetical protein AK89_04275) (6.25%), folding and protein processing (molecular chaperone DnaK) (6.25%), ribosomal structure (30S ribosomal protein S1) (6.25%) and stress (stress response regulator Gls24) (6.25%) (Table 1; Figure 5). These results indicate that the proteome of *Ent. mundtii* was affected by the presence of *E. coli* at 6 and 30 h although in a different way. In fact, a higher number of proteins were over produced at 30 h when the LAB achieved the stationary growth phase and the pathogen began its death cycle (Table 1; Figure 3C). Also, by performing a hypergeometric distribution, the probabilities of obtaining a certain COG category in our sample in relation with the ones encoded by the whole cell were analyzed. Whilst it was more probable to find one protein related with the metabolism and transport of carbohydrates (Figure S1A) we find five of them in our study (Table 1). This means that this category might be enriched by the obtained proteins. This also occurs with energy conversion and production, folding and protein processing and cell wall biosynthesis categories. For transcription, stress, ribosomal structure, and amino acid metabolism we found one of each as expected by the hypergeometric distribution. However, for the oxidoreductase (spot # 15) that includes three COG categories, was more probable to find two, and we found only one. This could imply an impoverishment of these categories (Figure S1A and Table 1).

#### Differential protein expression in *Ent. mundtii* CRL35 growing in co-culture at 6 and 30 h

Regarding the differential protein expression when *Ent. mundtii* grew in co-culture at T6 vs. T30, a total of 21 proteins were successfully identified. Twenty spots presented higher abundances at T6 than at T30 and one, the phosphoglucomutase/phosphomannomutase, was under expressed at T6. The overexpressed proteins participate of different functional categories, namely carbohydrate metabolism (phosphoglycerate mutase 1 family, glucose-6-phosphate isomerase, phosphoglycerate kinase, phosphopyruvate hydratase, phosphogluconate dehydrogenase, type I glyceraldehyde-3-phosphate dehydrogenase, transketolase) (40%), amino acid metabolism (glutamine synthetase, aminopeptidase, dipeptidase PepV and peptidase M13) (15%); energy production and conversion (ATP synthase subunit alpha, 2-oxoisovalerate dehydrogenase and F0F1 ATP synthase subunit beta) (20%), transcription (DNA-directed RNA polymerase subunit alpha) (5%), nucleotide metabolism (adenylosuccinate synthase) (5%), translation (leucine–tRNA ligase) (5%), stress (general stress protein) (5%), cell wall biosynthesis (choloylglycine hydrolase) (5%) (Table 2, Figure 5). Moreover, according to the hypergeometric distribution, the categories related with carbohydrate metabolism, energy production and conversion, amino acid transport and metabolism, nucleotide transport and metabolism are enriched. While the categories of translation, transcription, cell wall biogenesis, and of unknown function were obtained as expected (Figure S1B).

In the three proteome comparisons, higher number of differentially expressed proteins were related with carbohydrate, energy production, and amino acid metabolism (Figure 5).

### Functional analysis and interaction of proteins

**Figure 6:**
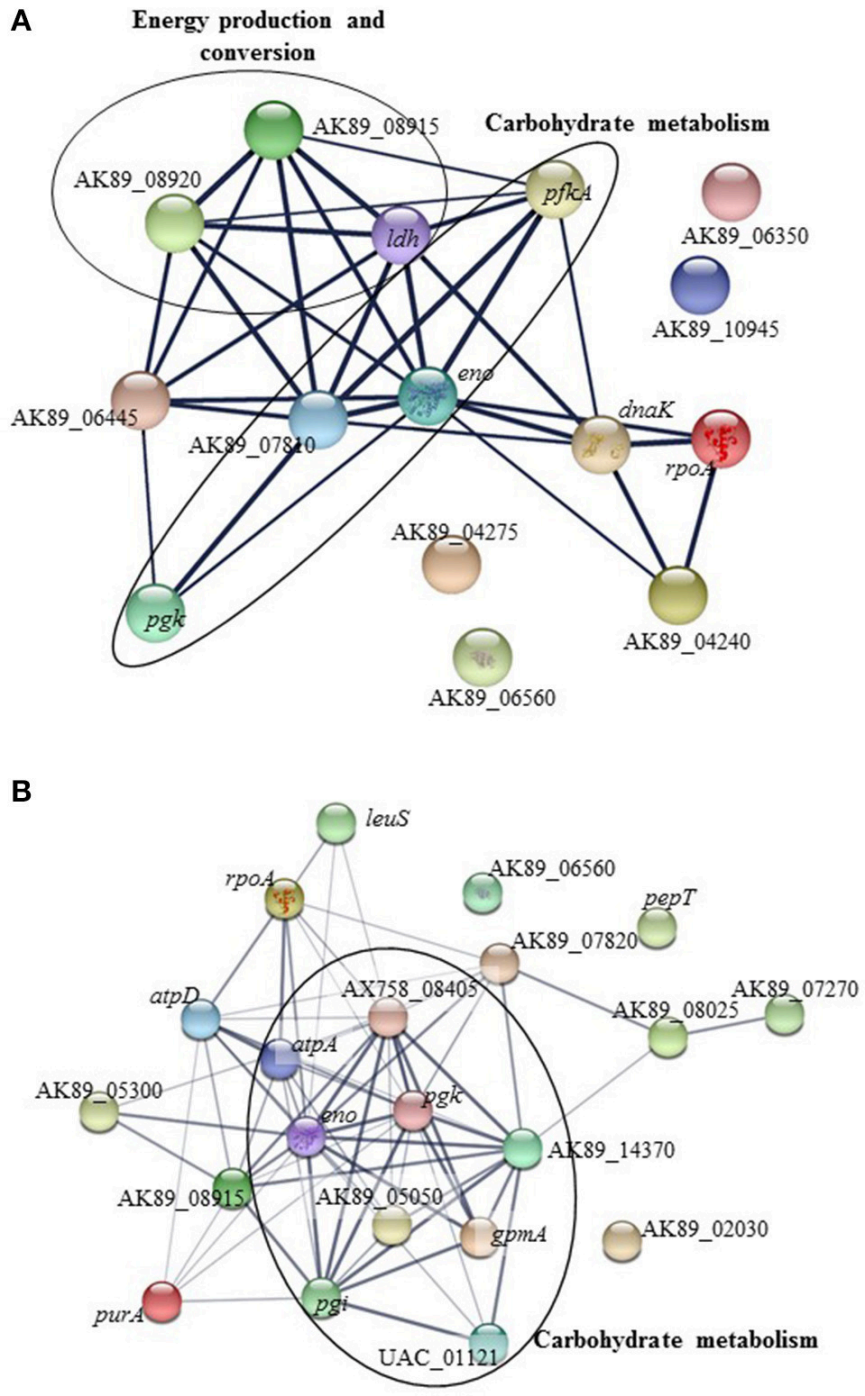
Protein-protein interaction network of overexpressed *Ent. mundtii* proteins. The circle highlights the proteins related with carbohydrate metabolism and energy production and conversion. The proteins are represented by nodes whereas their interactions by edges. Strength of the different interactions is represented by the thickness of the lines. The network was constructed with STRING v10.05. **(A)** Interaction network of *Ent. mundtii* proteins overexpressed in co-culture with respect to their individual grown at T30. **(B)** Interaction network of proteins overexpressed at T6 with respect to T30, when *Ent. mundtii* grew in co-culture with *E. coli*.

The interactions among the over expressed proteins from *Ent. mundtii* were obtained using the STRING v10.05 database. This analysis contributes to understand microbial performance in the meat-based medium. Two protein-protein interaction networks were constructed: (i) containing proteins over expressed by *Ent. mundtii* in co-culture with respect to their individual growth at T30 (Figure 6A). A network for T6 was not built as only four proteins at this time were over expressed and (ii) containing proteins over expressed at T6 with respect to T30 when *Ent. mundtii* was grown in co-culture with *E. coli* (Figure 6B). As shown in Figure 6A, 4 out of the 15 proteins over expressed at T30 with respect to the single culture at T30 have no interactions in between each other. However 11 proteins were related showing interactions (32 edges). Four proteins are related with sugar carbohydrate metabolism and four of them with energy production and conversion, which shows very strong interactions in between each other. This also support the fact that carbohydrates metabolism (G) through glycolysis is enriched at T30 in the presence of *E. coli*.

The second network (Figure 6B) corresponds to the overexpressed proteins of *Ent. mundtii* CRL35 grown in co-culture at T6 respect to their growth in co-culture at T30. Three out of 20 proteins were not included in the network as no interactions were found in the STRING database. In this network sixty eight interactions were obtained among the proteins overexpressed by *Ent. mundtii*, the thickness of lines between the nodes indicating the degree of interaction. As mentioned before, the overexpression of these 20 proteins in co-culture at T6 respect to T30 could be explained by the logarithmic growth phase of *Ent. mundtii* CRL35 at 6 h. Moreover, as it can be observed in Figure 6B, the main interactions found in the network are related with carbohydrate metabolism (9 proteins) in particular these involved in glycolysis and pentose phosphate pathway. This network support the activation and interaction of proteins related with carbohydrate metabolism in the exponential growth phase which is in agreement with the hypergeometric distribution test.

## Discussion

### Performance of LAB and *E. coli* NCTC 12900 in a meat-based medium. physiological results

The interaction and antagonistic activities of LAB with different pathogenic microorganisms were the focus of a number of studies. For instance, Atassi and Servin (2010) investigated the killing activity of *Lactobacillus* strains against *Salmonella enterica* serovar Typhimurium in co-cultures; Angmo et al. (2016) evaluated different LAB strains as biocontrol agents against *Yersinia enterocolitica* using agar spot tests as screening method. Thereafter, the growth of *Y. enterocolitica* in mixed cultures co-inoculated with two selected *Lactobacillus* strains was investigated. Also Yang et al. (2017) studied mixed cultures of bifidobacteria with *Listeria monocytogenes* to detect the changes in their growth pattern after mutual interaction by applying a proteomic approach. The present study is the first where the LAB inhibitory potential against *E. coli* O157:H7 is evaluated in co-cultures using physiological and proteomic approaches. The first objective of this study was to evaluate the inhibitory potential of three lactic acid bacteria strains toward *E. coli* O157:H7 NCTC12900. By a simple and rapid method such as the well-diffusion assay, a variable LAB inhibitory activity was evidenced. Then, the kinetic of growth in a meat model system in the presence of *E. coli*, was analyzed. The three LAB strains affected negatively the growth of EHEC in the meat environment after 8 h, evidencing a higher inhibitory potential of *L. plantarum* CRL681 and *Ent. mundtii* CRL35, which produced a significant decrease of *E. coli* counts at 96 h. It seems therefore that LAB trigged *E. coli* death, due to mechanisms other than acid effect or bacteriocin activity. In fact during the spot on lawn assay, no inhibitory halos were detected by supernatants containing acids or bacteriocins, they not triggering *E. coli* inhibition. Contrarily, Angmo et al. (2016) concluded that low pH and production of lactic acid were the main factors for inhibition of growth of *Y. enterocolitica* (Angmo et al., 2016). Moreover, even if all LAB strains were able to acidify the sarcoplasmic medium, they presented different inhibitory activity toward *E. coli*. According to this, EHEC has three powerful systems of resistance to acid stress; these including an acid-induced oxidative system, an acid-induced arginine-dependent system and a glutamate-dependent system (Bearson et al., 2009). These three systems of resistance to acids have different requirements, allowing an overlapping between them, ensuring that at least one of them will always be active to protect the cell in an acid environment (Bearson et al., 2009). These properties of EHEC contribute to low infectious doses by allowing small numbers of microorganisms to pass through the gastric acidity barrier. Therefore, its acid resistance ability is an important virulence factor and explains the absence of growth inhibition halos by culture supernatants observed herein. As regards to *E. coli* inhibition by bacteriocin action, it is known that these peptides do not act on Gram negative microorganisms unless they are combined with a treatment to damage the cell wall to allow bacteriocin entrance into the cell (Castellano et al., 2011). Therefore, our results, showing absence of inhibition due to *Ent. mundtii* CRL35 and *L. curvatus* CRL705 bacteriocins, suggest that EHEC antagonistic action involves other mechanisms such as competition for nutrients, quorum sensing, or a close cell-cell relationship where the bioprotective culture must preserve its vitality to cope with EHEC. In a similar work, Rios-Covian et al. (2015) reported a delayed growth of *Bacillus fragilis* by the presence of *Bifidobacterim longun* in co-culture during the first 14 h. They observed an improved growth of bifidobacteria compared to the corresponding mono-culture. Our results showed that *E. coli* affects slightly to moderately the maximal cell densities achieved by LAB. *Ent. mundtii* CRL35 showed an earlier exponential phase and the stabilization of the stationary phase with a slight viability decrease after 40 h compared to its growth in mono-culture. Yang et al. (2017) studying the co-incubation of *Bifidobacterium bifidum* with *Listeria monocytogenes*, have also reported the earlier entrance into the logarithmic growth phase suggesting a mutual growth promoting effect during the co-cultivation.

### Differential protein expression analyses

#### *Ent. mundtii* CRL35 in co-culture vs. *Ent. mundtii* in mono-culture

In this work, 2DE was employed to analyze differential expression induced by the interaction between *Ent. mundtii* CRL35 and *E. coli* NCTC12900, focusing on the LAB proteome. This approach allowed us to investigate the molecular basis of this interaction and the relation with the physiological changes undergoing during co-cultivation in the meat-based medium. Slight proteome variations were observed in *Ent. mundtii* during the first hours of co-culture with *E. coli* with respect to its growth as mono-culture at 6 h. One ABC transporter for methionine and the carbamoyl phosphate synthase large unit are among the over expressed proteins. They are related to amino acid biosynthesis and metabolism. One enzyme related to glycolysis, the enolase (spot #9), and one related to cell division (protein FtsZ, spot #14) resulted over expressed at 6 h and at 30 h indicating that LAB activated glycolysis and cell division to cope the presence of *E. coli*. It should be mentioned that enolase is also known as a moonlighting protein. These are proteins that display additional functions other than their major described biochemical catalytic activity. In general, these cytoplasmic/cell surface moonlighting proteins can be important in infection, virulence, or immune responses (Jeffery, 2015). For example enolase is also associated with epithelial cell binding (Castaldo et al., 2009). In fact, Peng et al. (2014) reported enolase as one of the actin-binding proteins in *Enterococcus faecalis*. It could therefore be suggested that *Ent. mundtii* can up-regulate enolase during co culturing with *E. coli* as an additional strategy to compete with *E. coli* for actin binding during adhesion to meat. On the other hand, *Ent. mundtii* proteome was much more affected in co-culture at 30 h than when it grew alone at the same time. In fact 16 proteins resulted over expressed, including some spots also up-regulated at 6 h (enolase and cell division protein FtsZ). Protein-protein network showed interaction in 12 of these proteins, mainly related with carbohydrate metabolism. The 31.25% of differentially over expressed proteins were involved in glycolysis. The 18.75% of proteins synthesized in higher amounts were related to other pathways, also involved in energy production and conversion, thus indicating that co-culturing with *E. coli* exerted more effective activation of these pathways at 30 h of co-culturing than during the first hours of the growth. In addition, physiological results indicate that at 30 h, *E. coli* is dying in co-culture, suggesting that *E. coli* viability decrease resulted convenient for *Ent. mundtii* which persisted in the stationary phase. *Ent. mundtii* during co-culture, resulted even more stable at stationary phase than when it grew alone. This fact is consistent with the over-expression of many proteins from sugar metabolism, energy production, transcription, cell division, and amino acid metabolism indicating an active metabolism of *Ent. mundtii* which allowed its persistence in the meat-based medium. It should also be highlighted the up regulation of proteins related to folding/processing and stress such as the chaperone DnaK and the stress response regulator Gls24 that could contribute to the satisfactory resistance of *Ent. mundtii* to stressful conditions dominating the microbial environment at 30 h in conjunction with a low pH (close to 4.0). There are some studies demonstrating the interaction of certain microorganisms during its growth in mixed cultures. Yang et al. (2017) proposed that the growth of *Bifidobacterium bifidum* WBBI03 and *Listeria monocytogenes* together promotes the growth of each other, resulting in earlier entry into the logarithmic phase, and the expression of proteins mostly tended to be up regulated at the translational and transcriptional level. While Rios-Covián et al. (2016) reported the stimulation of the growth of *Bifidobacterium longum* in co-culture while retarding the growth of *Bacteroides fragilis*, with concomitant changes in the production of some proteins and metabolites of both bacteria. In the present work a different interaction seems to occur between *Ent. mundtii* CRL35 and *E. coli* O157:H7. In fact, a positive effect of *E. coli* on the fitness of the LAB could occur, while the latter triggered the pathogen death after 8 h of co-culture.

#### *Ent. mundtii* CRL35 in co-culture: T6 vs. T30

**Figure 7:**
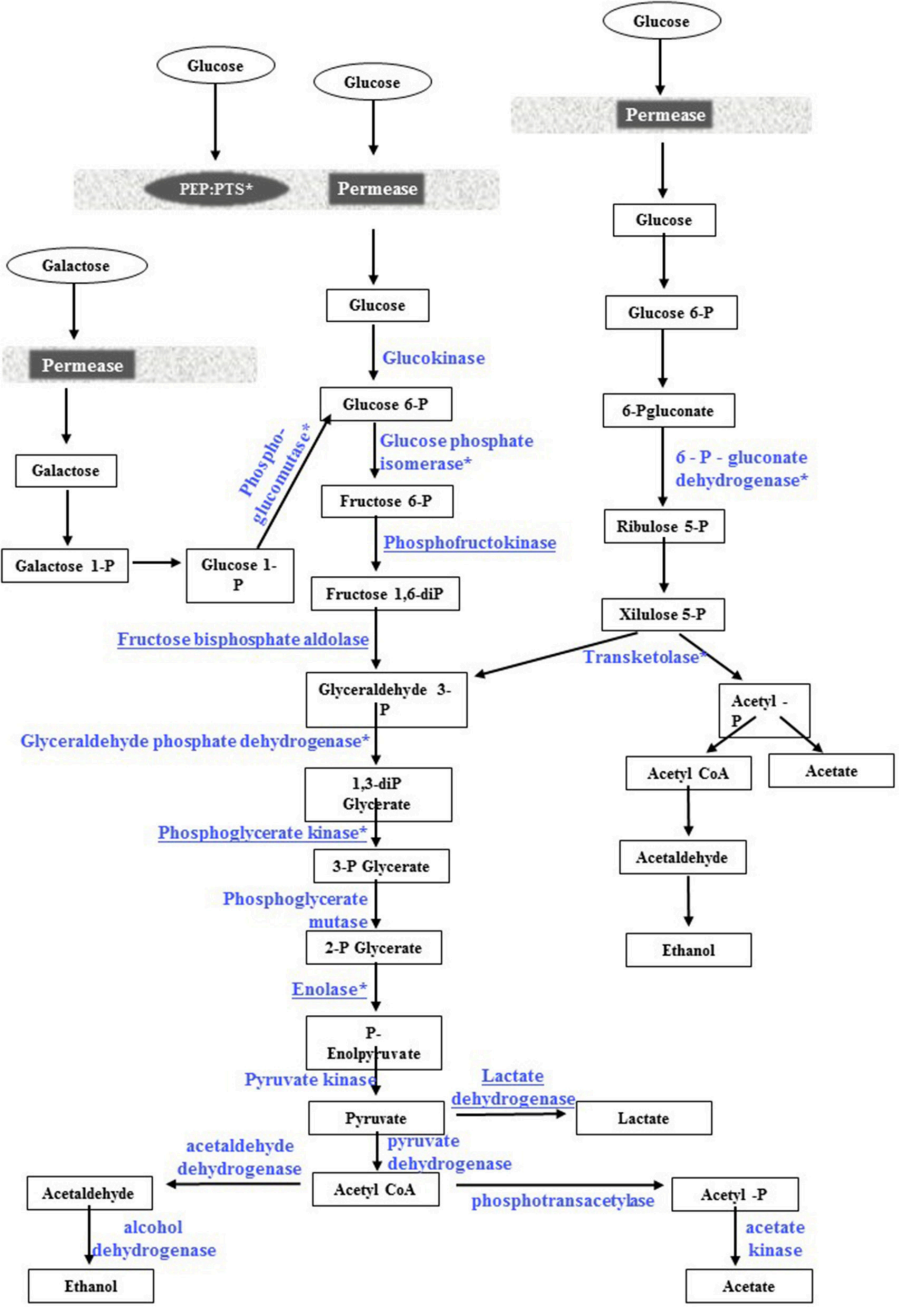
Glycolytic pathway in *Ent. mundtii* CRL35. Enzymes overexpressed at 30 h in co-culture with respect to their individual growth are underlined. With asterisks are presented the proteins overexpressed in co-culture at 30 h with respect to 6 h.

When comparing protein expression of co-cultures along the time (CC T6 vs. CC T30), 20 proteins resulted over expressed during the first hours (6 h). This is in relation to the exponential growth phase going through the microorganism, in which the general metabolism is activated as reported by other (Cohen et al., 2006; Koistinen et al., 2007). Those results are also supported by the observed protein network, where proteins related to carbohydrate metabolism presented stronger interactions. Among up regulated proteins, 7 spots were identified as belonging to carbohydrate metabolism and 1 to sugar transport. Five enzymes were related to glycolysis (spots #22, 23, 24, 25 and 27) and two involved in the pentose phosphate pathway (spots #26 and 28) (Figure 7). The over expression of phosphoenolpyruvate–protein phosphotransferase, involved in carbohydrate transport, could facilitate glucose entrance into the cell, as a consequence of a more efficient competition with the pathogen for sugar uptake, thus contributing to cope with *E. coli* presence which is also in the logarithmic growth state. Only one glycolytic enzyme, the phosphoglucomutase (spot #21) was under expressed at 6 h. This could be related to the up regulation of the PTS phosphoenolpyruvate-protein phosphotransferase (PTS system) involved in glucose transport by the generating glucose-6P which enters directly into the Embden-Meyerhorff-Parnas pathway, explaining the under expression of phosphoglucomutase which produces glucose-6P from glucose-1P coming from other pathways such as glycogen hydrolysis (Bonacina, 2017) (Figure 7). Glycogen metabolism would be less active than glucose during the first hours. In fact glucose is added to the meat-based medium which could be chosen firstly as primary energy source. On the other hand, two enzymes related to the pentose phosphate/phospohoketolase (PKP) pathway resulted over expressed in *Ent. mundtii* at 6 h, the 6-P gluconate dehydrogenase and transketolase, indicative also of the active metabolism of ribose, one of the sugars present in meat (Chaillou et al., 2005) (Figure 7). Concurring with our findings, Koistinen et al. (2007) reported that proteins preferentially expressed by *L. plantarum* in the early exponential phase were related to sugar consumption and biomass increase. Four enzymes related to amino acid metabolism were also up regulated by *Ent. mundtii* in co-culture at 6 h. Among them glutamine synthetase, a key enzyme of nitrogen metabolism that catalyzes the incorporation of ammonium into glutamate and is related to arginine biosynthesis, alanine, glutamate, and aspartate metabolism among other (Magasanik and Rothstein, 1980). In addition, some other peptidases and aminopeptidases resulted up regulated also at 6 h, indicating an active peptidolytic metabolism during the first hours of co-culturing. Also the adenylosuccinate synthase resulted 2.7-fold over expressed at 6 h with respect to T30 by *Ent. mundtii* in co-culture, this enzyme plays an important role in the *de novo* pathway of purine nucleotide biosynthesis, it catalyzing the first committed step in the biosynthesis of AMP from IMP, also indicating a more active metabolism of this LAB during the first hours of co-culture. Also, proteins involved in transcription and translation such as DNA-directed RNA polymerase subunit alpha, leucine-tRNA ligase resulted up regulated by this LAB strain during the first hours of co-culturing. In accordance, Yang et al. (2017), reported the up regulation of enzymes related to transcription and translation when mixed cultures of *Bifidobaterium bifidum* and *Listeria monocytogenes* were evaluated.

One general stress protein presented also higher amounts at T6, as well as, the choloylglycine hydrolase involved in lipid metabolism and cell wall biosynthesis. This pattern can be related with the exponential growth and the consequent active cellular division. Two ATP synthases (alpha and beta subunits) were found increased in this condition. They are the prime producers of ATP, using the proton gradient generated by oxidative phosphorylation. Finally, the 2-oxoisovalerate dehydrogenase beta subunit was 1.9 fold over expressed at 6 h during co-culture. This enzyme belongs to the oxidoreductase family, being also implicated in Phe, Tyr, Trp, Leu, Ile, Val, Asp, and Asn degradation. The branched-chain alpha-ketodehydrogenase complex catalyzes the overall conversion of alpha-keto acids to acyl-CoA and CO_2_. Summarizing, differential protein expression by *Ent. mundtii* in co-culture at two different growth phases correlated with the observed physiological behavior. The up regulation of many enzymes implicated in sugar and nitrogen metabolism, transcription, translation and energy production was in relation with the metabolism and the physiological state crossing LAB during the first hours of growth. A direct consequence of this active global metabolism contributed to the competition with *E. coli* at this specific moment when the pathogen was going through the exponential phase, while at 30 h, *Ent. mundtii* CRL35 reaching the stationary phase did not have to face a strong competition with *E. coli* since it had already entered into its death phase.

According with the obtained results, on can postulate the positive effect of *E. coli* on the fitness of the LAB, while a negative impact exerted the LAB on the pathogen by triggering its death after 8 h of co-culture. On the other hand, differential overexpression of *Ent. mundtii* proteins was higher in co-culture with *E. coli* than when it grew alone at 30 h. Concomitantly, physiological results indicated that at 30 h, *E. coli* was dying in co-culture, suggesting that the decrease of *E. coli* viability resulted convenient for *Ent. mundtii* which persisted in the stationary phase. This fact is consistent with the over-expression of many proteins from sugar metabolism, energy production, transcription, cell division and amino acid metabolism. This fact indicating the active *Ent. mundtii* metabolism allowed its persistence over the pathogen in the meat medium. It should also be highlighted the up regulation of proteins related to folding/processing and stress such as the chaperone DnaK and the stress response regulator Gls24 that could contribute to the satisfactory resistance of *Ent. mundtii* to stressful conditions at 30 h, such as the low pH. When comparing protein expression of *Ent. mundtii* in co-culture along the time (CC T6 *vs* CC T30), the higher number of proteins over expressed during the first 6 h, was in relation with the metabolism and the physiological state crossing LAB during the first hours of co-culture. A direct consequence of this active global metabolism contributed to the competition with *E. coli* which was going through the exponential phase. Whereas at 30 h, *Ent. mundtii* was reaching the stationary phase and *E. coli* had already entered into its death phase. Indeed the LAB did not have to face a strong competition, accordingly, less differential protein expression was achieved at 30 h. Finally, mechanisms involved in this interaction, such as competition for nutrients, quorum sensing, or a close cell-cell relationship are suggested. The detailed study of these mechanisms is focus of ongoing investigations.

## Conclusion

Current results have demonstrated the true inhibitory potential of LAB against a pathogen of great concern such as *E. coli* O157:H7. Such inhibition was not due to acid or bacteriocin production but instead to a more complex relationship during the microbial interaction. The proteomic results herein presented supported physiological observations, demonstrating significant differences in protein expression in LAB (i) due to the presence of the pathogen and (ii) according to the growth phase analyzed. Even when more studies have to be performed in fresh meat to confirm *in vitro* observations, these results lay the foundations of the molecular basis for understanding the interaction between *Ent. mundtii* CRL35 and *E. coli* O157:H7 NCTC12900, as well as on the strategies of competition applied by both microorganisms. This work finally opens new perspectives for the application of this bioprotective LAB to control *E. coli* O157:H7 in meat products.

## Author contributions

AO carried out the experiments, analyzed the results and wrote the paper. SF conceived the idea of the project, coordinated the study, analyzed and discussed the results and wrote the paper. MS coordinated the study, discussed the results and wrote the paper. LT performed the *in silico* analysis of the identified proteins (STRING, COGNITOR) and the analysis of the hypergeometric distribution. JR and AA contributed in mass spectrometric analysis (MALDI-TOF/TOF) and discussed the paper. GV contributed to the discussion of the paper. All authors read and approved the final manuscript.

### Conflict of interest statement

The authors declare that the research was conducted in the absence of any commercial or financial relationships that could be construed as a potential conflict of interest.

## References

[B1] Altschul S. F. Gish W. Miller W. Myers E. W. Lipman D. J. (1990). Basic local alignment search tool. J. Mol. Biol. 215, 403–410. 10.1016/S0022-2836(05)80360-2 2231712

[B2] Angmo K. Kumari A. Bhalla T. C. (2016). Antagonistic activities of lactic acid bacteria from fermented foods and beverage of Ladakh against *Yersinia enterocolitica* in refrigerated meat. Food Biosci. 13, 26–31. 10.1016/j.fbio.2015.12.004

[B3] Atassi F. Servin A. L. (2010). Individual and co-operative roles of lactic acid and hydrogen peroxide in the killing activity of enteric strain *Lactobacillus johnsonii* NCC933 and vaginal strain *Lactobacillus gasseri* KS120. 1 against enteric, uropathogenic and vaginosis-associated pathogens. FEMS Microbiol. Lett. 304, 29–38. 10.1111/j.1574-6968.2009.01887.x 20082639

[B4] Bearson B. L. Lee I. S. Casey T. A. (2009). *Escherichia coli* O157: H7 glutamate- and arginine-dependent acid-resistance systems protect against oxidative stress during extreme acid challenge. Microbiology 155(Pt 3), 805–812. 10.1099/mic.0.022905-0 19246751

[B5] Belfiore C. Castellano P. Vignolo G. (2007). Reduction of *Escherichia coli* population following treatment with bacteriocins from lactic acid bacteria and chelators. Food Microbiol. 24, 223–229. 10.1016/j.fm.2006.05.006 17188201

[B6] Best A. La Ragione R. M. Cooley W. A. O'Connor C. D. Velge P. Woodward M. J. (2003). Interaction with avian cells and colonisation of specific pathogen free chicks by Shiga-toxin negative *Escherichia coli* O157:H7 (NCTC 12900). Vet. Microbiol. 93, 207–222. 10.1016/S0378-1135(03)00031-2 12695045

[B7] Bonacina J. (2017). Genomic and Functional Analysis of Enterococci Isolated From Food. Doctoral thesis, Universidad Nacional de Tucumán.

[B8] Bustos A. Y. de Valdez G. F. Raya R. de Almeida A. M. Fadda S. Taranto M. P. (2015). Proteomic analysis of the probiotic *Lactobacillus reuteri* CRL1098 reveals novel tolerance biomarkers to bile acid-induced stress. Food Res. Int. 77, 599–607. 10.1016/j.foodres.2015.10.001

[B9] Candiano G. Bruschi M. Musante L. Santucci L. Ghiggeri G. M. Carnemolla B.. (2004). Blue silver: a very sensitive colloidal Coomassie G-250 staining for proteome analysis. Electrophoresis 25, 1327–1333. 10.1002/elps.200305844 15174055

[B10] Castaldo C. Vastano V. Siciliano R. A. Candela M. Vici M. Muscariello L.. (2009). Surface displaced alfa-enolase of *Lactobacillus plantarum* is a fibronectin binding protein. Microbes Cell Fact. 8:14. 10.1186/1475-2859-8-14 19220903 PMC2654425

[B11] Castellano P. Belfiore C. Vignolo G. (2011). Combination of bioprotective cultures with EDTA to reduce *Escherichia coli* O157: H7 in frozen ground-beef patties. Food Control 22, 1461–1465. 10.1016/j.foodcont.2011.02.018

[B12] Chaillou S. Champomier-Vergès M.-C. Cornet M. Crutz-Le Coq A.-M. Dudez A.-M. Martin V.. (2005). The complete genome sequence of the meat-borne lactic acid bacterium *Lactobacillus sakei* 23K. Nat. Biotechnol. 23, 1527–1533. 10.1038/nbt1160 16273110

[B13] Chikindas M. L. Weeks R. Drider D. Chistyakov V. A. Dicks L. M. (2017). Functions and emerging applications of bacteriocins. Curr. Opin. Biotechnol. 49, 23–28. 10.1016/j.copbio.2017.07.011 28787641 PMC5799035

[B14] Cohen D. P. Renes J. Bouwman F. G. Zoetendal E. G. Mariman E. de Vos W. M.. (2006). Proteomic analysis of log to stationary growth phase *Lactobacillus plantarum* cells and a 2-DE database. Proteomics 6, 6485–6493. 10.1002/pmic.200600361 17115453

[B15] Colello R. Caceres M. E. Ruiz M. J. Sanz M. Etcheverria A. I. Padola N. L. (2016). From farm to table: follow-up of shiga toxin-producing *Escherichia coli* throughout the pork production chain in Argentina. Front. Microbiol. 7:93. 10.3389/fmicb.2016.00093 26903972 PMC4744844

[B16] De Man J. Rogosa D. Sharpe M. E. (1960). A medium for the cultivation of lactobacilli. J. Appl. Microbiol. 23, 130–135.

[B17] Fadda S. Sanz Y. Vignolo G. Aristoy M. Oliver G. Toldra F. (1999). Hydrolysis of pork muscle sarcoplasmic proteins by *Lactobacillus curvatus* and *Lactobacillus sakei*. Appl. Environ. Microbiol. 65, 578–584. 9925585 10.1128/aem.65.2.578-584.1999 PMC91064

[B18] Fadda S. Vignolo G. Holgado A. P. Oliver G. (1998). Proteolytic activity of Lactobacillus strains isolated from dryfermented sausages on muscle sarcoplasmic proteins. Meat Sci. 49, 11–18. 10.1016/S0309-1740(97)00097-1 22063180

[B19] Fadda S. Vildoza M. J. Vignolo G. (2010). The acidogenic metabolism of *Lactobacillus plantarum* CRL 681 improves sarcoplasmic protein hydrolysis during meat fermentation. J. Muscle Foods 21, 545–556. 10.1111/j.1745-4573.2009.00202.x

[B20] Galperin M. Y. Makarova K. S. Wolf Y. I. Koonin E. V. (2015). Expanded microbial genome coverage and improved protein family annotation in the COG database. Nucleic Acids Res. 43(Database issue), D261–D269. 10.1093/nar/gku1223 25428365 PMC4383993

[B21] Grosu-Tudor S.-S. Brown L. Hebert E. M. Brezeanu A. Brinzan A. Fadda S.. (2016). S-layer production by *Lactobacillus acidophilus* IBB 801 under environmental stress conditions. Appl. Microbiol. Biotechnol. 100, 4573–4583. 10.1007/s00253-016-7355-5 26910041

[B22] Jeffery C. J. (2015). Why study moonlighting proteins? Front. Genet. 6:211. 10.3389/fgene.2015.00211 26150826 PMC4473056

[B23] Koistinen K. M. Plumed-Ferrer C. Lehesranta S. J. Karenlampi S. O. von Wright A. (2007). Comparison of growth-phase-dependent cytosolic proteomes of two *Lactobacillus plantarum* strains used in food and feed fermentations. FEMS Microbiol. Lett. 273, 12–21. 10.1111/j.1574-6968.2007.00775.x 17559397

[B24] Lim J. Y. Yoon J. Hovde C. J. (2010). A brief overview of *Escherichia coli* O157:H7 and its plasmid O157. J. Microbiol. Biotechnol. 20, 5–14. 20134227 PMC3645889

[B25] Magasanik B. Rothstein D. M. (1980). The role of glutamine synthetase in the regulation of bacterial nitrogen metabolism, in Glutamine: Metabolism, Enzymology, and Regulation, eds Mora J. Palacios R. (Mexico: Elsevier), 61–68.

[B26] Nally J. E. Grassmann A. A. Planchon S. Sergeant K. Renaut J. Seshu J.. (2017). Pathogenic leptospires modulate protein expression and post-translational modifications in response to mammalian host signals. Front. Cell. Infect. Microbiol. 7:362. 10.3389/fcimb.2017.00362 28848720 PMC5553009

[B27] Peng Z. Krey V. Wei H. Tan Q. Vogelmann R. Ehrmann M. A.. (2014). Impact of actin on adhesion and translocation of *Enterococcus faecalis*. Arch. Microbiol. 196, 109–117. 10.1007/s00203-013-0943-1 24362949

[B28] Pingitore E. V. Todorov S. D. Sesma F. de Melo Franco B. D. G. (2012). Application of bacteriocinogenic *Enterococcus mundtii* CRL35 and *Enterococcus faecium* ST88Ch in the control of *Listeria monocytogenes* in fresh Minas cheese. Food Microbiol. 32, 38–47. 10.1016/j.fm.2012.04.005 22850372

[B29] Rios-Covián D. Sánchez B. Martínez N. Cuesta I. Hernández-Barranco A. M. de los Reyes-Gavilán C. G.. (2016). A proteomic approach towards understanding the cross talk between *Bacteroides fragilis* and *Bifidobacterium longum* in coculture. Can. J. Microbiol. 62, 623–628. 10.1139/cjm-2015-0804 27156738

[B30] Rios-Covian D. Sánchez B. Salazar N. Martínez N. Redruello B. Gueimonde M.. (2015). Different metabolic features of *Bacteroides fragilis* growing in the presence of glucose and exopolysaccharides of bifidobacteria. Front. Microbiol. 6:825. 10.3389/fmicb.2015.00825 26347720 PMC4539542

[B31] Saavedra L. Minahk C. de Ruiz Holgado A. P. Sesma F. (2004). Enhancement of the enterocin CRL35 activity by a synthetic peptide derived from the NH2-terminal sequence. Antimicrob. Agents Chemother. 48, 2778–2781. 10.1128/AAC.48.7.2778-2781.2004 15215149 PMC434193

[B32] Salvucci E. Saavedra L. Sesma F. (2007). Short peptides derived from the NH2-terminus of subclass IIa bacteriocin enterocin CRL35 show antimicrobial activity. J. Antimicrob. Chemother. 59, 1102–1108. 10.1093/jac/dkm096 17449885

[B33] Szklarczyk D. Franceschini A. Wyder S. Forslund K. Heller D. Huerta-Cepas J.. (2015). STRING v10: protein-protein interaction networks, integrated over the tree of life. Nucleic Acids Res. 43(Database issue), D447–D452. 10.1093/nar/gku1003 25352553 PMC4383874

[B34] UniProt Consortium (2015). UniProt: a hub for protein information. Nucleic Acids Res. 43, D204–D212. 10.1093/nar/gku989 25348405 PMC4384041

[B35] Varsha K. K. Nampoothiri K. M. (2016). Appraisal of lactic acid bacteria as protective cultures. Food Control 69, 61–64. 10.1016/j.foodcont.2016.04.032

[B36] Vignolo G. Castellano P. Fadda S. (2015). Starter cultures: bioprotective cultures, in Handbook of Fermented Meat and Poultry, 2nd Edn., eds Todrá F. Astiasaran Y. H. Hui I. Sebranek J. G. Talon R. (Malden, MA: Blackwell Publishing Inc.), 129–137.

[B37] Wessels S. Axelsson L. Hansen E. B. De Vuyst L. Laulund S. Lähteenmäki L. (2004). The lactic acid bacteria, the food chain, and their regulation. Trends Food Sci. Technol. 15, 498–505. 10.1016/j.tifs.2004.03.003

[B38] Woraprayote W. Malila Y. Sorapukdee S. Swetwiwathana A. Benjakul S. Visessanguan W. (2016). Bacteriocins from lactic acid bacteria and their applications in meat and meat products. Meat Sci. 120, 118–132. 10.1016/j.meatsci.2016.04.004 27118166

[B39] Yang D. Wu X. Yu X. He L. Shah N. P. Xu F. (2017). Mutual growth-promoting effect between *Bifidobacterium bifidum* WBBI03 and *Listeria monocytogenes* CMCC 54001. J. Dairy Sci. 100, 3448–3462. 10.3168/jds.2016-11804 28259400

