# Supplementary figures and images for: Differential Proteomic Analysis of Lactic Acid Bacteria—Escherichia coli O157:H7 Interaction and Its Contribution to Bioprotection Strategies in Meat
Source: Front Microbiol. 2018 Jun 5;9:1083. doi: 10.3389/fmicb.2018.01083 (PMC5996242; doi:10.3389/fmicb.2018.01083)

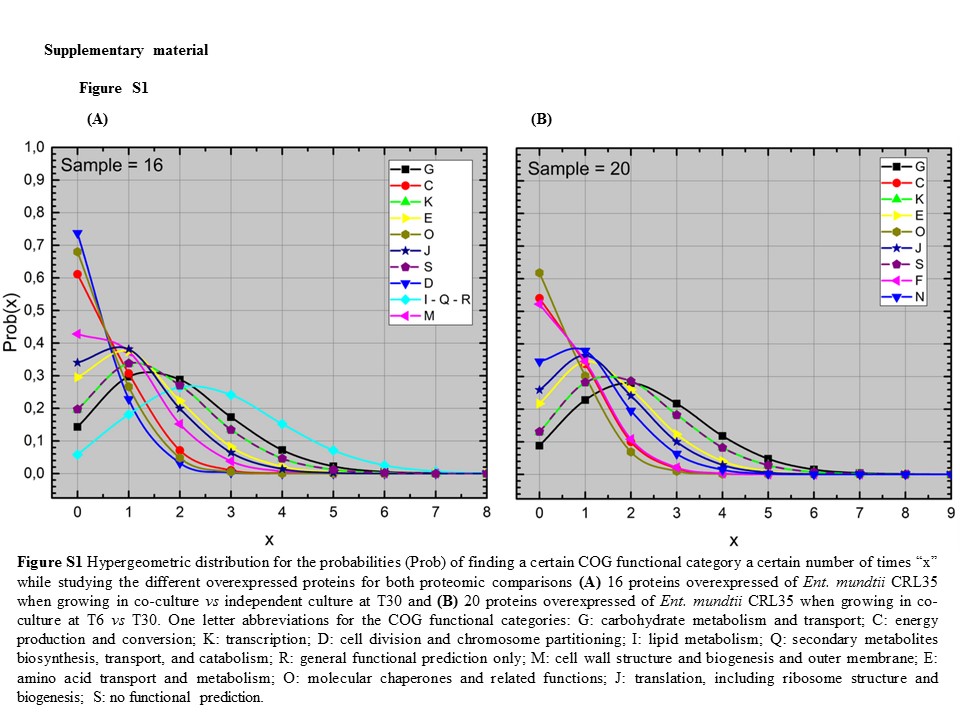

Supplement: Supplementary file 1 [file Image_1.JPEG]
